# The Role of Se-Containing Glutathione Peroxidases and Thioredoxin Reductases in Oncogenesis: Expression Paradoxes and Therapeutic Prospects

**DOI:** 10.3390/antiox15030312

**Published:** 2026-03-01

**Authors:** Elena G. Varlamova, Sergey V. Gudkov, Egor A. Turovsky

**Affiliations:** 1 Institute of Cell Biophysics of the Russian Academy of Sciences, Federal Research Center “Pushchino Scientific Center for Biological Research of the Russian Academy of Sciences”, 142290 Pushchino, Russia; 1928lv@mail.ru (E.G.V.); turovsky.84@mail.ru (E.A.T.); 2 Prokhorov General Physics Institute of the Russian Academy of Sciences, 38 Vavilove St., 119991 Moscow, Russia; 3 Department of Fundamental Sciences, Bauman Moscow State Technical University, 5, 2nd Baumanskaya St., 105005 Moscow, Russia

**Keywords:** selenoproteins, glutathione peroxidase, thioredoxin reductase, redox signaling, cancer, dual role, tumor suppressor, oncogene, ferroptosis, therapy resistance

## Abstract

This review synthesizes current evidence on the dualistic and context-dependent roles of selenium-containing antioxidant enzymes—specifically, glutathione peroxidases (GPXs) and thioredoxin reductases (TXNRDs)—in the development and progression of human cancers. We analyze how these crucial components of cellular redox homeostasis can function as either potent oncogenes or tumor suppressors depending on the tissue of origin, cancer stage, genetic background, and tumor microenvironment. The paradoxical behavior of these enzymes is governed by a complex interplay of transcriptional regulation, epigenetic modifications, and signaling pathway interactions, ultimately influencing critical processes such as apoptosis, proliferation, invasion, and therapy resistance. Special emphasis is placed on the unique role of GPX4 in regulating ferroptosis, a promising target for novel anti-cancer strategies, and on the prognostic significance of TXNRD overexpression in aggressive malignancies. By integrating data across various cancer types, this review highlights these enzyme families as central molecular switches in carcinogenesis and discusses their potential as biomarkers and targets for rational, combination-based therapeutic interventions.

## 1. Introduction

It is well known that imbalances in redox balance in cancer cells result in elevated levels of reactive oxygen species (ROS), a class of highly reactive molecules closely associated with the pathogenesis of various cancers. The dual role of ROS in cancer biology is that at moderate concentrations, they promote cancer progression, while at high levels, they can lead to damage and death of cancer cells. ROS generation is regulated by a complex set of subtle molecular mechanisms, including the modulation of the expression of oncogenes, tumor suppressors, and redox enzymes [1].

The thioredoxin and glutaredoxin systems play a key role in regulating the redox balance in healthy and cancer cells. These systems exhibit a variety of biological activities, including protection against oxidative stress and regulation of DNA synthesis, the cell cycle, and apoptosis [2]. The key enzymes in these systems are thioredoxin reductases (TXNRDs) and glutathione peroxidases (GPXs). It is well known that various types of cancer cells experience increased synthesis of the tripeptide glutathione, which may be due to increased expression of key enzymes of the γ-glutamyl cycle, as well as changes in the transport rate of its precursors [3]. Members of the GPX family utilize glutathione as a reducing agent in the reduction of hydrogen peroxide or organic peroxides. In mammals, Se-containing and non-Se GPXs are distinguished, which significantly influences the expression levels of these enzymes in cancers of various etiologies and their mechanism of action to maintain redox balance. Se-containing members of the thioredoxin system are thioredoxin reductases, which are enzymes of the triphosphopyridine nucleotide (NADPH) oxidative pathway and play a key role in several physiological processes such as redox pathways and DNA synthesis [4].

This broad antioxidant network, however, acquires a unique layer of complexity and therapeutic significance when focusing on its selenium (Se)-dependent members. Unlike non-Se antioxidant enzymes (e.g., Cu/Zn-superoxide dismutase, catalase) or small-molecule scavengers (e.g., vitamins C and E), the activity of GPX1-4, GPX6, and TXNRD1-3 is critically dependent on the trace element selenium [5,6]. Selenium is co-translationally incorporated into the active site of these enzymes in the form of the 21st amino acid, selenocysteine (Sec) [7]. The selenol group (-SeH) in Sec is a superior nucleophile compared to the thiol group (-SH) of cysteine, making these enzymes exceptionally efficient catalysts for redox reactions [6]. This provides them with a unique kinetic advantage in rapidly neutralizing peroxides and regulating redox signaling. This dependency on selenium creates a profound biochemical paradox. On one hand, adequate selenium intake is essential for the synthesis and activity of these tumor-suppressive enzymes [8,9,10]. On the other hand, the same machinery can be hijacked by cancer cells; the upregulation of selenoprotein synthesis supports their survival under high oxidative stress and confers resistance to therapy [11,12]. This delicate balance dictates that the role of these enzymes is not merely antioxidant but rather context-dependent, functioning as a “rheostat” that can either suppress malignant transformation or fuel tumor progression [11,13]. Understanding the unique selenocysteine-dependent mechanisms of these enzymes is therefore paramount to deciphering their dichotomous roles in oncogenesis.

This review aims to elucidate the role of Se-containing glutathione peroxidases and thioredoxin reductases in oncogenesis, specifically, to investigate the dichotomous role of their expression in cancers of various etiologies. We propose that the dual expression pattern of the same enzyme in different types of cancer cells or in different cell lines of the same cancer may be explained not only by different ROS levels in these cells, but also by the dichotomous nature of the Se they contain. In recent decades, sufficient evidence has accumulated to indicate that the trace element Se plays a significant role in carcinogenesis, with its effects being dual in nature. This is due not only to the narrow range of its therapeutic concentrations but also to the nature of the Se-containing agent.

It is known that Se is a key component of a wide range of organic and inorganic compounds for which an anti-cancer effect has been proven [14,15,16,17,18,19,20,21,22,23,24]. It also plays an important role and is necessary for the synthesis of selenoproteins involved in the regulation of many important body processes, including cancer [25,26,27,28,29,30,31,32,33]. Furthermore, exogenous administration of certain selenoproteins, such as selenoprotein V, has been shown to directly trigger apoptosis in cancer cells by inducing mitochondrial dysfunction and ROS overproduction [34]. Having a huge amount of data on the involvement of selenoproteins in the progression or inhibition of carcinogenesis of various etiologies, it is important to systematize the available information on the expression levels of these proteins in various types of cancer cells and identify possible patterns of such regulation. In this review, we will focus on the consideration of Se-containing glutathione peroxidases and thioredoxin reductases, establish patterns of their expression in various tumor cells or tissues, and explore how the nature of their expression determines their key role as an oncogene or oncosuppressor.

A critical aspect of understanding the dichotomous nature of these selenoproteins lies in adopting a stage-specific and context-dependent framework. The function of a given enzyme is not static but evolves during the multistep process of carcinogenesis. During the tumor initiation phase, the primary role of antioxidant selenoproteins like GPX1 and TXNRD1 is often tumor-suppressive. They protect normal cells from oxidative DNA damage and mutation, thereby preventing malignant transformation. However, once a tumor is established, cancer cells undergo a process of “redox adaptation.” They upregulate the very same antioxidant systems—now functioning as proto-oncogenic factors—to manage their own intrinsically high levels of reactive oxygen species (ROS), resist apoptosis, and survive in the hostile tumor microenvironment. This shift from a protective to a pro-survival, pro-growth function is central to the “expression paradox.” In this review, we will not only catalog the dual roles of GPX and TXNRD isoforms but also attempt to map their functions onto this temporal axis of cancer progression, from initiation to advanced, therapy-resistant disease.

## 2. The Nature of the Expression of Se-Containing Glutathione Peroxidases in Cancers of Various Etiologies and Signaling Pathways with Their Participation

The Se-dependent glutathione peroxidase (GPXs) family is central to the regulation of cellular redox homeostasis by catalyzing the reduction of peroxides by glutathione. Human GPX consists of eight isoenzymes (GPX1-8), of which five members (GPX1-4 and 6) are selenocysteine (Sec)-containing proteins, and three members (GPX5, 7 and 8) are cysteine-containing proteins in which the Sec active site is replaced with cysteine. These isoenzymes are encoded by different genes and differ in their molecular structure, subcellular localization, substrate specificity, enzyme properties, and biological function [5].

### 2.1. Glutathione Peroxidase 1 (GPX1)

The most well-studied and widespread is the GPX1 isoform, which is expressed sequentially [35,36]. Analysis of current data on GPX1 mRNA expression patterns in cancer cells of various etiologies has revealed surprising paradoxes, and polymorphisms of the gene of this enzyme have complex associations with cancer risks and patient survival [8,11,37]. At the transcriptional level, GPX1 expression is regulated by several transcription factors: PU.1, p53, NF-κB, AP-1, AP-2, ZNF143, OREBP [38,39,40,41,42]. To date, there is sufficient evidence that GPX1 is involved in both pro- and anti-cancer effects in various tumor models. GPX1 is closely related to tumor development, patient survival, and prognosis in various human malignancies, however, expression levels and prognostic values of GPX1 in various types of tumors are still controversial. The following is an overview of the latest data on the expression and activity of GPX1, as well as the regulation of its gene expression at the molecular level in cancers of various origins.

Different patterns of GPX1 expression are observed in breast cancer [43]. At the molecular level, GPX1 expression is regulated in various breast cancer cell lines by the transcription factor TFAP2C (AP-2γ), which binds to the 5′-GCCNNNGGC-3′ region in the GPX1 promoter in the absence of CpG methylation in this region. It was found that in BT-474 and MDA-MB-453 cancer cells, methylation of the GPX1 promoter region is practically absent, therefore AP-2γ modulates GPX1 expression, enhancing it. Whereas in MCF-7 and SKBR3 cells, extensive methylation of the GPX1 promoter region is observed, which prevents AP-2γ from binding to the GPX1 promoter region; therefore, the expression of this enzyme is reduced [44]. The transcription factor AP-2γ is known to be a key regulator of hormone sensitivity in breast carcinoma cells through the control of multiple estrogen signaling pathways, including direct binding to the estrogen receptor-α (ERα) promoter [45]. When upregulated, GPX1 helps prevent cell cycle arrest [46] and significantly increases drug resistance in breast cancer cells [43,47]. On the other hand, Jablonska et al. showed that breast cancer risk was significantly associated with the GPX1 rs1050450 (Pro198Leu) polymorphism, indicating a protective effect of the leucine (Leu) variant allele [48]. It was shown that 36% of GPX1 breast cancer tissues with the Leu-containing allele had low enzymatic activity of this glutathione peroxidase [49].

GPX1 is strongly expressed in renal cell carcinoma (ccRCC) cells, which accounts for 80–90% of kidney malignancies, and correlates with a number of pathological processes in ccRCC tissues and the stage of this disease [50]. Thus, using various independent approaches, it was found that both mRNA and GPX1 protein expression in kidney cancer cell lines A-498, ACHN, 786-O, CAKI-1 is much higher than in normal kidney epithelial cells GK-2. Unfortunately, there is currently no information about the molecular mechanisms regulating GPX1 expression during ccRCC progression.

It was found that patients with head and neck squamous cell carcinoma (HNSCC) have a high ratio of disulfide glutathione to reduced glutathione (GSSG/GSH). Such patients had a high level of oxidative stress and a high risk of recurrence of the local tumor after treatment [51]. It was found that GPX1, an antioxidant capable of oxidizing glutathione (GSH), is overexpressed in HNSCC cells [52,53,54]. It was found that the ligand 16 of the CXC chemokine motif (CXCL16) in HNSCC correlates with an unfavorable prognosis, as well as with the proliferation, migration and invasion of tumor cells. Recently, in the work of He and co-authors, CXCL16 can influence the growth of HNSCC cells by regulating the antioxidant pathway GPX1 [53]. On the other hand, a significant negative correlation between the HNSCC stage and GPX1 expression was found in the work of Dequanter and co-authors [55]. In addition, the authors established a link between GPX1 expression and tumor localization: patients with low GPX1 expression had more tumors located in the tonsils.

It was also found that GPX1 expression correlated with clinical manifestations, lymph node metastases, and overall survival in patients with laryngeal squamous cell carcinoma (LSCC) [56]. High expression of GPX1 also promotes invasion, migration, proliferation, and cisplatin resistance of esophageal squamous cell carcinoma (ESCC) [57,58]. It was shown that high expression of GPX1 was observed in two ESCC lines (EC9706 and EC109) and low in two (K150 and K180 cell lines) [58]. In this work, the authors identified the molecular mechanisms of regulation of GPX1 expression through vitamin D/NF-κB/GPX1. It was also found that increased expression of GPX1 stimulates migration and invasion through matrix metalloproteinase-2 MMP2 (MMP2) and urokinase-type plasminogen activator uPA, which are critical accelerators of tumor invasion and migration processes [59,60,61].

In addition, a signaling pathway triggered by hepatocyte growth factor (HGF) in gastric cancer cell lines such as NUGC3 and MKN28, mediated by NF-κB and GPX1, has been identified [62]. The increase in GPX1 levels under the action of HGF and its regulation by NF-κB emphasize its importance in the progression of gastric cancer. In addition, GPX1 expression was influenced by the PI3K/Akt pathway, as well as uPA expression and activity. On the other hand, the presence of low levels of GPX1 mRNA and promoter methylation was found in the gastric cancer cell lines SNU1 and SNU484 [63]. Loss of GPX1 expression was significantly associated with advanced gastric cancer and lymphatic invasion, but not with lymph node metastases, Loren classification, or vascular invasion. Overexpression of GPX1 significantly correlated with lymph node metastases, widespread general stage, depth of invasion > 10 mm, high degree of malignancy and perineural invasion in oral squamous cell carcinoma (OSCC) cells [64]. Relapses were more common in the group with high GPX1 expression, whereas the 5-year survival after surgery was 70.6% in the group with low GPX1 expression and 59.6% in the group with high GPX1 expression. Thus, in patients with high GPX1 expression, the prognosis of relapse and survival was unfavorable.

Increased expression of GPX1 has also been shown to promote cell proliferation, invasion, migration, and resistance to cisplatin, as well as enhance apoptosis in salivary adenoid cystic carcinoma (SACC) cells [65]. The authors found that the NF-κB pathway is associated with proliferation, cisplatin resistance, invasion, migration, and apoptosis in SACC through positive regulation of GPX1 expression and uPA activation.

In hepatocellular carcinoma (HCC) cells, GPX1 expression levels directly depended on the level of Se-binding protein 1 (SBP1), which enhanced GPX1 activity and suppressed hypoxia-induced factor-1a (HIF-1a), contributing to the invasiveness of HCC [66]. It was found that SBP1 and GPX1 physically interact, and under conditions of oxidative stress are able to form nuclear corpuscles and colocation. GPX1 is also capable of suppressing SBP1 transcription. On the other hand, we previously showed that GPX1 expression increased significantly in HepG2 cells after 48 h of exposure to the multikinase inhibitor sorafenib, a well-known anti-cancer drug of the first lineage [37].

Different patterns of GPX1 expression have been recorded for different types of brain cancer. In brain tumors, including glioblastomas and neuroblastomas, GPx1 overexpression is usually observed, which contributes to multidrug resistance and invasiveness [67,68]. It has also been shown that GPX1 is expressed at a higher level in glioblastomas than in low-grade gliomas. The authors found that exosomes secreted by glioblastoma cells overexpress GPX1, which increases the resistance of cancer cells to peroxide and radiation. Also, high levels of GPX1 expression in glioblastoma can critically increase the levels of immune infiltration of B cells, CD4^+^, macrophages, neutrophils, and dendritic cells. In low-grade gliomas, a positive correlation was observed between GPX1 mRNA expression and the level of CD8^+^ T cell infiltration [69]. High expression of GPX1 in glioblastomas promotes hypoxic catabolism of hydrogen peroxide and inhibition of apoptosis by activating HIF1a [70]. Another proof that GPX1 contributes to the progression of glioblastoma multiforme was the study of post-transcriptional modifications of this enzyme, namely, aberrant splicing of its pre-mRNA [71]. Thus, an octamer-binding protein that does not contain the POU domain (NONO), which is overexpressed in human gliomas, participates in the splicing of GPX1 pre-mRNA, which contributes to the production of normal GPX1 glioblastoma cells.

GPX1 plays a complex role in the development of colon cancer. It was found that an increase in GPX1 expression correlated with late stages (III–IV), low differentiation, lymph node and distant metastases in colorectal cancer [11]. Statistical calculations revealed a relationship between the accumulation of GPX1 in pathologically altered colon tissue and the degree of malignancy of the tumor [72]. On the other hand, low expression of the GPX1 gene may be common in colon cancer, indicating a dichotomous role for this gene. In vitro analysis of GPX1 expression in colorectal cancer cell lines showed that the highest level of GPX1 protein expression was found in the HCA-2 cell line, while the lowest level of expression was observed in the SW1116 cell line. GPX1 was identified as a protein associated with a decrease in 5-year survival in patients with colon adenocarcinoma on based on the results of the Cox regression model [73]. In addition, GPX1 expression was associated with the expression of proliferating cell nuclear antigen (PCNA), which is commonly present in proliferating cells and tumor cells and is directly related to cellular DNA synthesis, the degree of tumor differentiation, and tumor prognosis [74,75].

GPx1 expression negatively correlates with the prognosis of pancreatic ductal adenocarcinoma (PDAC), acting as a tumor suppressor and inhibiting cancer cell proliferation. GPX1 has been shown to inhibit the epithelial–mesenchymal transition (EMT) and chemoresistance by regulating the Akt/GSK3β/Snail signaling axis in PDAC [76]. The Akt/GSK3β/Snail pathway is well known to be a signaling cascade that regulates the transcription factor Snail, which is critical for EMT. In this pathway, AKT phosphorylates GSK-3β (glycogen synthase kinase-3 beta), inactivating this enzyme. This leads to Snail activation, which promotes EMT and cancer progression [77].

Information on the levels and mechanisms of regulation of GPX1 expression in cancers of various etiologies is summarized in Table 1.

### 2.2. Glutathione Peroxidase 2 (GPX2)

Analysis of GPX2 expression patterns reveals a more unambiguous pro-oncogenic focus [78,79,80,81,82,83,84,85,86]. This enzyme is primarily expressed in the cytosol of mammary gland and gastrointestinal cells, as well as in human liver [13,87,88]. The GPX2 promoter harbors the antioxidant/electrophile response element (ARE/EpRE), which is highly conserved in mice, rats, and humans. It has been identified in the GPX2 promoter and has been shown to be a target of the nuclear factor E2/Kelch-like ECH-associated protein 1 (Nrf2/Keap1) system. GPX2 is a direct target of the transcription factor PU.1.

Complex patterns of GPX2 expression are observed in breast cancer. Thus, a dramatic decrease in the expression levels of this glutathione peroxidase was found in the highly metastatic PyMT2 tumor cell line compared to the poorly metastatic PyMT1 tumor cell line [89]. Furthermore, PyMT1 cells produced lower levels of ROS than PyMT2 cells. These data thus suggest a direct correlation between low GPX2 expression, reduced ROS generation, and malignant transformation. Further examination of breast cancer subtypes revealed that GPX2 mRNA was reduced in HER2-enriched compared to luminal A and B tumors, as well as in basal-like tumors, implying a link between GPX2 loss and pathological progression. Activation of hypoxia, Notch, and NF-kB signaling pathways was observed in low-expression GPX2 subtypes of breast cancer. Overall, the authors conclude that suppression of GPX2 expression has a broad effect on tumor phenotype, which is consistent with oncogenic ROS signaling. A more thorough analysis revealed that suppression of GPX2 expression stimulated malignant progression due to activation of ROS/HIF1a/VEGFA signaling, causing poor perfusion and hypoxia [89].

On the contrary, there is work that shows a direct correlation between the level of GPX2 expression and breast cancer progression, which was demonstrated on ER-positive MCF-7 cells and ER-negative MDA-MB231 cells [90]. The authors explain this by the fact that as soon as the cells are programmed for uncontrolled proliferation, GPX2 supports the growth of cancer cells and inhibits apoptosis [91]. Another study also demonstrated that the suppression of GPX2 apoptosis caused by oxidative stress in MCF-7 depended on wild-type p53 [92]. Similar conclusions were drawn in the work of Naiki-Ito et al. [93], who analyzed breast cancer cell lines with both wild-type p53 (C2 and MCF-7) and mutant p53 (C11 and T47D) to investigate any link between GPX2 and p53. The authors found that a decrease in GPX2 expression caused inhibition of cell proliferation in breast carcinoma C2 and MCF-7 cell lines with wild type p53, but not with the p53 mutation. Therefore, the GPX2 gene may be a target for breast cancer therapy using wild-type p53, especially in the early stages of breast carcinogenesis, including precancerous conditions.

It has recently been shown that GPX2 increases in patients with non-small cell lung cancer and promotes proliferation, migration, invasion, tumor growth, and cisplatin resistance of non-small cell lung cancer cells with KRAS mutation [94]. It was found that GPX2 activation can be an early event in the development of lung tumors, and high expression of this selenoprotein gene is associated with low overall survival in patients with lung adenocarcinoma. The authors found that overexpression of GPX2 reduces the accumulation of ROS and increases the expression of matrix metalloproteinase-1 (MMP1) in NSCLC cells with the KRAS mutation. In addition, GPX2 has been shown to directly affect miR-325-3p, whereas overexpression of miR-325-3p reverses the effects of GPX2 in NSCLC cells with KRAS mutation [94]. Peng et al. showed that GPX2 was highly expressed in NSCLC cells, but was practically absent in neighboring normal tissues, with high GPX2 levels associated with poorer overall survival in patients with NSCLC, positive lymph node metastases, stage TNM I or stage TNM III [78]. When investigating the potential mechanism by which GPX2 promotes tumor metastasis, it was found that the PI3K/AKT signaling pathway is an important downstream pathway regulated by GPX2 in A549 cells. It was found that overexpression of GPX2 increased the levels of phosphorylation of PI3K, AKT, and mTOR, while knockdown of GPX2, on the contrary, decreased them. The results obtained indicate that GPX2 can reduce the level of ROS in NSCLC cells, as well as promote the epithelial–mesenchymal transition. Similar results were also obtained in the work of Derakhshan Nazari et al., who reported that an increase in GPX2 activity during the final progression of the tumor to stage IV correlates with the pathway of glutathione metabolism, which supports the redox balance in cancer cells against accumulating reactive oxygen species (ROS) and, apparently, promotes EPT, which leads to metastasis [95]. The authors also found that GPX2 in the final stages of NSCLC provides cancer cells with their excess glucose levels, which strongly affects the metabolism of cancer cells, providing them with energy through intensive glycolysis. Unfortunately, there is no data on the suppressive role of GPX2 in lung cancer.

It has also been repeatedly demonstrated that GPX2 acts as a cancer marker for glioblastomas. Thus, a comparative analysis of GPX2 mRNA expression was performed in healthy patients and patients with glioblastoma, depending on gender, age, race, and survival rate [96]. The results of the analysis showed that male patients were characterized by a direct correlation between an increase in GPX2 mRNA expression and poor survival, a similar situation was observed for Caucasian patients, while an inverse relationship was established for African Americans. In addition, GPX2 has been shown to co-express enzymes involved in the chemokine signaling pathway (CXCR1, CXCR2, and XCR1). Recently, there has been an increasing number of studies proving the essential role of chemokines and their receptors in cellular interactions associated with inflammation and carcinogenesis [97,98,99]. There is also a publication demonstrating that GPX2 is one of the three favorable genes positively associated with the overall survival of glioblastoma patients, along with COX10, which encodes cytochrome c oxidase and COMT, which encodes catechol-O-methyltransferase [100]. Thus, this is an example of GPX2 being positively associated with overall patient survival, indicating that protection against oxidative damage may exist in CNS tumors.

It is believed that GPX2 can also serve not only as an effective biomarker for predicting HCC, but also as a prognostic tool for identifying patients with stage I according to the TNM classification who have a high risk of recurrence. The co-expression of GPX2 and alphafetoprotein (AFP) is a reliable criterion for identifying patients with high-risk HCC after hepatectomy, which can help in determining the optimal treatment strategy [90]. In a broader context, a recent in vivo study using a TAA-induced mouse model of HCC demonstrated significant dysregulation of mRNA expression of several selenoproteins during both fibrotic and tumorigenic stages, underscoring the complexity of selenoproteome regulation during liver cancer progression [101]. The Kaplan–Meier analysis revealed that patients with higher GPX2 levels had a correlation with lower levels of overall and relapse-free survival [77]. In addition, the results showed that GPX2 suppression significantly increased ROS levels in HepG2 cells, while GPX2 overexpression significantly reduced ROS levels in another HCC Huh7 cancer cell line. Moreover, the authors showed that inhibition of GPX2 expression can sensitize HepG2 cells to lenvatinib, while Huh7 cells with overexpression of GPX2 became more resistant to lenvatinib. Based on the data obtained, a mechanism was suggested according to which lenvatinib induces ROS-associated apoptosis by regulating the β-catenin/GPX2 axis through direct interaction of β-catenin/TCF4 with the GPX2 promoter in HepG2 cells [77,102]. Current data have revealed a potential mechanism of action of lenvatinib and demonstrated its ability to inactivate β-catenin in HCC [77]. In continuation of this study, Tan et al. have been demonstrated that lenvatinib, by inhibiting GPX2 expression in vivo and in vitro, can induce the development of immunogenic cell death, regulatory cell death, triggering adaptive immune responses directed against dead cell antigens. In addition, the authors have shown that GPX2 inhibition significantly enhances the antitumor efficacy of lenvatinib, causing endoplasmic reticulum stress [103]. To date, no data has been recorded regarding the suppressive role of GPX2 in HCC.

GPX2 also plays the role of a cancer marker in gastric cancer of various etiologies. Among all the gastric cancer cell lines studied, GPX2 expression levels were particularly high in gastric carcinoma NUGC-4 and MKN-45 cells compared with normal gastric epithelial cells GES-1, MKN-74, AZ-521, MKN-1. The authors proved that GPX2 plays an important role in prognosis and progression [104]. Thus, knockdown suppressed proliferation, invasion, migration, and epithelial–mesenchymal transition in cancer cells in vitro and in vivo. In addition, proteomic analysis showed that GPX2 expression regulates metabolism mediated by kynureninase (KYNU), which is a key protein in tryptophan catabolism by cleaving the tryptophan metabolite kynurenine (kyn), which is an endogenous ligand for the aryl hydrocarbon receptor (AhR). This receptor controls critical biological processes (cell differentiation, apoptosis, and cell metastasis). In addition, the authors found that activation of the ROS-mediated KYNU-kyn-AhR signaling pathway caused by GPX2 knockdown is involved in the progression of gastric cancer and metastasis [104]. In another study, it was found that GPX2 is also a prognostic marker for diffuse gastric cancer, which is characterized by high malignancy and frequency of metastasis, as well as poorly understood etiology, which leads to unfavorable outcomes for patients [105]. Using an integrated approach, the authors proved the key role of GPX2 in the progression of diffuse gastric cancer by regulating lipid metabolism. Thus, functional suppression of GPX2 disrupts the formation of lipid droplets and lipid homeostasis, which leads to an increase in the level of acylcarnitine, which disrupts the function of mitochondria. In addition, disruption of lipid homeostasis is synergistic with endoplasmic reticulum stress, which eventually triggers apoptosis in gastric cancer cells. In particular, GPX2 inhibition increases the effectiveness of cisplatin by sensitizing cancer cells to apoptosis. These data identify GPX2 not only as an important prognostic biomarker, but also as a promising therapeutic target for overcoming cisplatin resistance in diffuse gastric cancer [105].

In colorectal cancer, elevated GPX2 levels serve as a specific marker, peaking in adenomas and adenocarcinomas, where it is associated with advanced stages, lymphovascular invasion, and decreased survival [106]. It was also shown that GPX2 knockout mice developed fewer tumors and foci with depleted mucin caused by the action of azoxymethane than wild-type mice. At the same time, slowing down the development of tumors may be associated with the effective elimination of damaged or precancerous epithelial cells in mice with GPX2 knockdown. The expression level of GPX2 was increased in wild-type dysplastic crypts, where it appears to act antiapoptotically and thus support tumor development [107]. The potential dual role of GPX2 in carcinogenesis was studied in human colon adenocarcinoma cells, HT-29 clones, in which the expression of this enzyme was stably reduced, leading to high expression of cyclooxygenase COX-2 [108]. It was found that GPX2 clearly inhibits cell migration and invasion, obviously counteracting the expression/activity of COX-2, but at the same time promotes the growth of tumor cells. Thus, manipulations with GPX2 can be both harmful and beneficial, depending on the stage of tumor development [109].

Low level of GPX2 mRNA expression is a common process in the progression of bladder cancer, which is closely associated with an unfavorable prognosis in patients with pT2-4 urothelial carcinomas [110]. In addition, low levels of GPX2 expression correlated with advanced stages of ulcerative colitis. It was also found that a decrease in GPX2 expression in patients with ulcerative colitis of the bladder may be associated with an early stage of cancer invasion [111]. In addition, decreased GPX2 expression indicates an unfavorable prognosis in patients with urothelial carcinomas of the upper urinary tract and bladder [112]. On the other hand, it was found that the expression of this enzyme gradually increased with progression from normal to papillary or nodular hyperplasia and urothelial carcinoma. After subcutaneous transplantation of BC31 cells with a low GPX2 content to mice, an increase in apoptotic cancer cell death was observed, and the inhibition of angiogenesis was largely due to the induction of apoptosis, as well as inhibition of angiogenesis and squamous cell differentiation. These results indicate that GPX2 may be one of the most important biomarkers or therapeutic targets in bladder cancer [113].

A direct correlation between the level of GPX2 expression and the progression of carcinogenesis has also been established for prostate cancer. Thus, inhibition of GPX2 expression can significantly reduce the levels of proliferation and invasion of LNCaP and 22RV1 cancer cells and enhance the apoptotic death of these cells [86]. The authors have established that the mechanism of regulation of GPX2 processes associated with the progression of prostate cancer is associated with the Wnt/β-catenin/EPT signaling pathway. The authors also found that GPX2 has a certain correlation with antitumor immunity, since the level of GPX2 expression can affect the participation of eight types of immune cells in the immune response in prostate cancer. In another study, the role of GPX2 in the regulation of castration-resistant prostate cancer was investigated [81]. The authors established a direct correlation between the level of GPX2 expression and the progression of the disease: suppression of GPX2 expression contributed to the inhibition of cell growth in castration-resistant cancer of rat (PCai1 line) and human (PC3 line), which was due to cyclin-B1 dependent cell cycle arrest in the G2/M phase. Thus, these results again indicate that GPX2 is a prognostic marker of castration-resistant prostate cancer.

It was also shown that GPX2 was significantly overexpressed in patients with squamous cell carcinoma of the head and neck [114]. In this work, it was found that the regulation of GPX2 expression in FaDu and Detroit 562 cancer cells depended on NRF2, which enhanced it, which was accompanied by cancer progression. When GPX2 was inhibited, there was a significant decrease in cell proliferation, a decrease in the size of 3D culture spheres, and an inhibition of migration and invasion, but an increase in ROS and the proportion of cells with apoptosis was observed. In addition, at the molecular level, a decrease in GPX2 expression reduced the signaling of Hedgehog (HH) and NOTCH, which play a key role in tumor formation [115,116]. Thus, the authors found that NRF2 regulates the progression of squamous cell carcinoma of the head and neck through the NRF2-GPX2-NOTCH3 axis.

Information on the levels and mechanisms of regulation of GPX2 expression in cancers of various etiologies is summarized in Table 2.

### 2.3. Glutathione Peroxidase 3 (GPX3)

The main extracellular isoform of GPXs is GPX3, expressed predominantly by the parietal cells of the Bowman capsule and the proximal tubular epithelium in the kidneys [117,118,119,120,121,122,123]. In addition, GPX3 is localized in the thyroid gland [124], in the epididymis [125], and binds to the basement membranes of the lungs and intestines [126]. Thus, most of the GPX3 in plasma is expressed by the kidneys and partially by other organs. In addition to Se-dependent translation, this enzyme is also regulated at the transcriptional level. Thus, it is regulated by PPARy (the gamma receptor activated by peroxisomal proliferator) [127,128,129], and contains binding sites for HIF-1 (hypoxia-induced factor 1), Sp1 (protein specificity 1), MRE (metal response element), and ARE (antioxidant response element) [130]. In addition, GPX3 expression can be regulated epigenetically by hypermethylation of the promoter [131,132,133,134], deletion in the gene of this enzyme [135], and by missense mutations in the coding region [136,137].

It is well known that GPX3 is subject to epigenetic modifications, in particular, methylation of the promoter, which is accompanied by its dysfunction, and therefore its expression is significantly reduced in many tumors. For example, Zhu et al. found that GPX3 was hypermethylated in 22 samples from 24 tumor tissues of esophageal squamous cell carcinoma, which was significantly associated with M and N stages of cancer, but had no relation to gender, age, history, and tumor size [136,137,138,139]. In order to understand whether GPX3 can act as a tumor suppressor, the authors overexpressed GPX3 mRNA in various esophageal squamous cell carcinoma lines. It was found that GPX3 is able to suppress proliferation, metastasis, and MMP-9 expression by deactivating the FAK (focal adhesion kinase)/AKT (protein kinase B) signaling pathway.

Other authors have also found that hypermethylation of the GPX3 promoter is observed in gastric cancer cells [138,140]. Based on the available clinical and pathological information, it was found that hypermethylation of GPX3 in gastric cancer cells, accompanied by a decrease in its expression, significantly correlated with lymph node metastases [139,141]. In addition, GPX3 expression has been shown to be significantly reduced in gastric adenocarcinoma STAD tissues compared to normal gastric tissues, and patients with high GPX3 expression have better long-term survival rates, suggesting a potential tumor-suppressive function of GPX3 [140,142]. On the other hand, He et al. showed that a decrease in GPX3 expression was associated with a more favorable prognosis in patients with gastric cancer, while an increase in GPX3 expression was significantly associated with tumor progression [141,143]. However, GPX3 has a tumor-suppressing effect during the transition from the normal epithelial mucosa to the early stage of gastric cancer, and at the same time, GPX3 can promote the development of tumors in cells that have undergone malignant transformation, protecting them from apoptosis. The work showed the regulation of GPX3 expression by binding to miR-502-3p, which prevents its translation or promotes degradation. In addition, the authors identified a potential regulatory pathway for the transformation of the normal gastric mucosa into gastric cancer: DUBR/miR-502-3p/GPX3, where DUBR (DPPA2 Upstream Binding RNA). In addition, GPX3 may contribute to the development of gastric cancer by increasing the infiltration of tumor immune cells, enhancing the expression of immune checkpoints, and reducing sensitivity to chemotherapeutic drugs [141,143]. It was also found in gastric cancer models that GPX3 selectively inhibits the Wnt/JNK signaling pathway compared to the canonical Wnt/β-catenin pathway via NFkB [142,144].

High expression of GPX3 in ovarian tumor samples, including subtypes of highly malignant serous and clear cell cells, has been reported by many authors [145,146,147,148,149]. It was found that GPX3 is necessary for optimal tumor growth in vivo in a syngenetic model of ovarian cancer, and that the expression of pro-oncogenic and immunomodulatory growth factor GDF15, a mitokine that has proliferative and metastatic functions, depends on the expression of GPX3 [145]. Other authors have found that samples of ovarian cancer patients are divided into two groups according to the level of GPX3 expression, while increased GPX3 expression is associated with a higher stage of the disease and lower overall patient survival. In addition, GPX3 is necessary for the survival of HGSA ovarian cancer cells in the ascitic environment of the tumor and protects against the extracellular effects of oxidative stress. This may indicate that GPX3 is an important factor in adaptation to transcelomic metastasis [146]. DNA microarray analysis revealed that GPX3 is highly expressed in clear cell adenocarcinoma of the ovaries, whereas suppression of GPX3 by RNA interference increased sensitivity to cisplatin by 3.3–4.2 in cancer cells [147]. Screening of ovarian cancer cell lines allows us to identify several HGSA cell lines with high basal GPX3 expression or with induced GPX3 expression during matrix detachment.

Epigenetic regulation of GPX3 activity is a common phenomenon in colorectal cancer cells. It was found that hypermethylation of the GPX3 promoter region was observed in about one third of colorectal cancer cell samples, which significantly reduced its expression and increased cell sensitivity to oxaliplatin and cisplatin [150]. The results showed that the expression of the cholesterol-related gene GPX3 was negatively associated with cholesterol levels, but positively correlated with the Ki-67 proliferation index in colorectal cancer [151]. GPX3 expression was higher in patients with low-grade and late-stage colorectal cancer and high cholesterol.

On the other hand, it was found that overexpression of GPx3 significantly suppressed the proliferation, migration, and invasion of lung cancer cells and stopped growth in the G2/M phase. In addition, the expression of cyclin B1 (a downstream modulator of cell cycle arrest) specifically decreased with the expression of GPX3, which regulated this process through the MKP3-Erk-NF-κB-cyclin B1 signaling cascade by deactivating ROS [152]. It has also been shown that overexpression of GPx3 significantly reduces the nuclear translocation of NF-kB under oxidative stress, thereby suppressing cyclin B1 expression in lung cancer cells. Li et al. also showed that low expression of GPX3 is observed in lung adenocarcinoma cells, while the authors investigated the role of opsin 3, a member of the superfamily of G-protein coupled receptors, in the progression of lung adenocarcinoma [153]. As a result of this study, an inverse correlation was established between the expression of GPX3 and opsin 3, which allowed the authors to assume that opsin 3 stimulates the progression of this disease by resolving redox homeostasis through the GPX3 pathway. In other work, it was also proved that GPX3 is poorly expressed in cells and tissues of non-small cell lung cancer [154]. The authors investigated the molecular mechanism of regulation of cancer progression through studying the expression levels of circular RNA circ_0078767, miR-665 and GPX3. As a result, a chain of interactions was established: miR-665 is the target of circ_0078767, whereas overexpression of miR-665 can significantly reduce the expression of GPX3 circ_0078767, which indicates that GPX3 is a downstream target for miR-665, which inhibits its expression. Thus, circ_0078767 modulates the behavior of NSCLC cells through the miR-665/GPX3 pathway. Another study demonstrates the involvement of miR in the regulation of GPX3 in lung cancer, which found that miR-196a in non-small cell lung cancer cells is involved in the progression of NSCLC by regulating the Jun N-terminal kinase (JNK) pathway by targeting GPX3 [155]. The results showed that suppression of miR-196a expression and restoration of GPX3 expression suppressed the viability, proliferation, and self-renewal of cancer cells. In addition, decreased miR-196a expression and, conversely, high GPX3 expression could reduce the degree of phosphorylation of JNK and c-Jun. In the paper by Choi et al. the relationship between GPX3 and miR-92 in A549 lung cancer cells was also investigated by interacting miR-92 with a site in the 3′-untranslated region of GPX3 and inhibiting its expression [156]. In the work of other authors, the analysis of the GPX3 gene predicted the presence of ten elements of the glucocorticoid response (GRE) in its promoter [157]. When studying the effect of the corticosteroid dexamethasone (Dex) on GPX3 expression, a direct correlation was established between the expression levels of GPX3 and Dex, which significantly induced the expression of the studied glutathione peroxidase in the cell lines H1299, H1650 and H1975. Initially, these cancer cell lines showed a low level of GPX3 expression due to hypermethylation of the promoter region of its gene. As a result of this study, the authors proved that GPx3 expression can be independently regulated through epigenetic or GR-mediated mechanisms in lung cancer cells, and suggest that GPx3 may enhance anti-inflammatory signaling mediated by glucocorticoids (GC) in lung cancer cells.

When studying GPX3 expression levels in breast cancer cells, Lou et al. showed that this glutathione peroxidase, due to its high expression, can be considered as a biomarker for the diagnosis and prognosis of breast cancer [158]. Thus, the authors not only found that increased expression of GPX3 significantly suppressed the proliferation, migration, and invasion of breast cancer cells, but also identified two potential mechanisms responsible for reducing its expression: hypermethylation of the promoter and the release of the miR-324-5p inhibitor. Initially, mRNA expression and relative GPX3 content were significantly reduced in two breast cancer cells MCF-7 and MDA-MB-231 compared with the healthy MCF-10A cell line, as well as in breast cancer tissues compared with neighboring healthy tissues. In addition, low GPX3 expression significantly negatively correlated with estrogen/progesterone expression and positively correlated with tumor size, histopathological grade of malignancy, and lymph node metastases. All these data suggest that GPX3 is negatively correlated with breast cancer progression and may act as a tumor suppressor in this disease. A similar effect of inverse correlation of GPX3 expression level with breast cancer progression was documented in studies by Saelee et al. [159]. The authors found that in most of the breast cancer samples studied, a decrease in GPX3 expression was recorded, which correlated with the number of metastatic nodes and the size of the tumor. When studying the role of epigenetic modification of GPX3 in inflammatory breast cancer, it was found that hypermethylation of the GPX3 promoter is widespread in these tissues compared with healthy ones [160]. Low levels of GPX3 expression in inflammatory breast cancer tissues contribute to the progression of carcinogenesis by cancer invasion into lymphatic vessels, formation of tumor emboli and increased chemoresistance. On the other hand, GPX3 expression was increased in more invasive TNBC triple negative breast cancer cells, which caused these cells to become resistant to cisplatin by increasing TGFß1 expression [161]. The authors identified the GPX3-TGFB1-ZEB2 regulatory axis in TNBC progression.

Hypermethylation of the GPX3 promoter is also common in human papillary thyroid cancer, which is associated with tumor size and metastasis to lymph nodes [162]. In this study, it was found that thyroid cancer metastasis is suppressed by GPX3 through inhibition of the Wnt/β-catenin signaling system. In another study, it was also proved that GPX3 expression is significantly suppressed in thyroid cancer cells, however, significant tumor suppressive activity was observed along with JUN overexpression of this protein. This suggests that GPX3 and JUN may act as important suppressor genes in thyroid cancer [163]. It was also found that lower GPX3 levels were associated with a higher malignancy of thyroid cancer, as GPX3 levels were significantly lower in stage IV thyroid cancer than in stages I, II and III [164]. In addition, thyroid cancers with BRAFV600E mutations were shown to have lower GPX3 levels. It was also found that miR-146b-5p negatively regulates GPX3 expression in thyroid cell lines. miR-146b-5p is known to be overexpressed in papillary thyroid carcinoma, especially in combination with the BRAFV600E mutation. This contributes to the growth of lymph node metastases, extrathyroid invasion, and clinical stage thyroid cancer.

Information on the levels and mechanisms of regulation of GPX3 expression in cancers of various etiologies is summarized in Table 3.

### 2.4. Glutathione Peroxidase 4 (GPX4)

The gene GPX4 contains 8 exons, and its expression is regulated by several transcription factors including Sp1, TFAP2A/AP2 (transcription factor AP-2 α) [165]. In addition, posttranslational modifications play an important role in the regulation of GPX4 expression and activity, for example, ubiquitination, phosphorylation, succination, and aclylation [166,167]. This glutathione peroxidase, unlike others, is most often a target in the development of drugs for the treatment of cancer of various etiologies. Numerous studies demonstrate that GPX4 inhibitors increase sensitivity to chemotherapy, radiotherapy, and immunotherapy, and lead to the activation of ferroptosis in cancer cells [168,169,170,171,172,173,174,175,176,177,178]. Ferroptosis is a programmed cell death or iron-catalyzed form of regulated necrosis, which is characterized by the accumulation of ROS and excessive formation of lipid peroxidation (POL).

It has been reported that glioblastomas exhibit a higher sensitivity to ferroptosis compared to other types of cancer [179,180]. The expression of GPX4 in glioblastomas and the mechanisms of its inhibition are described in detail in [178]. Here we briefly list the main GPX4 inhibitors, which contributed to the growth of ferroptosis and cancer cell death. For example, orexin-A, a multifunctional neuropeptide, contributed to a decrease in GPX4 expression in glioblastoma cells, causing ferroptosis mediated by lipid peroxidation (POL) [181]. In another study, it was shown that the GPX4 inhibitor could be plumbagin, which reduced only the level of protein, but not GPX4 mRNA, and the drug was able to cause GPX4 degradation through the lysosomal pathway [182]. In addition, GPX4 is a critical target in glioblastoma cell death during dihydroartemisinin treatment [182,183]. It was also shown that increased expression of GPX4 disrupted the activation of the NFkB signaling pathway, which was corrected by treating glioblastoma cells with RAS-selective lethal 3 (RSL3) inhibitor [183,184]. A nanoconstruction based on iron nanoparticles was also developed, which effectively delivered iron, cisplatin, and small interfering GPX4 RNA to cancer cells, which ultimately enhanced ferroptosis [184,185]. Another example of the fact that GPX4 inhibition stimulates ferroptosis in glioblastoma cells is the therapeutic effect of fatostatin [185,186], after treatment with which a decrease in the expression levels of AKT (protein kinase B), mTOR, 4EBP1 (protein 1 binding translation initiation factor 4E) was observed in glioblastoma cells, the authors concluded that inhibition GPX4 synthesis by fatostatin is mediated by activation of the AKT/mTORC1/4EBP1 signaling pathway, which, in turn, contributed to the death of GB cancer cells by ferroptosis. Beyond GPX4, manipulating the expression of other selenoproteins, such as SELENOM, has been shown to modulate the sensitivity of glioblastoma cells (A-172 line) to ER stress-inducing agents, further demonstrating the complex role of the selenoproteome in GBM biology [187]. There is also a study that provides important information about the anti-cancer properties of borax by suppressing the HSPA5/NRF2/GPx4/GSH signaling pathway and activating ferroptosis in glioblastoma cells [188]. In addition, it was found that FOXP3 (a member of the FOX family of transcription factors) can bind to five sites in the GPX4 promoter, which indicates direct regulation of FOXP3 transcription of GPX4 [189]. Thus, FOXP3 binds to the promoter and activates GPX4 transcription, which leads to a decrease in the level of ferroptosis and, ultimately, to the proliferation of glioblastoma cells. In another study, it was shown that the curcumin analog ALZ003 induced apoptosis and ferroptosis, characterized by an increase in the activity of caspase and LPO, respectively, and associated with the accumulation of ROS, as well as by suppressing GPX4 [190]. Transcription factor NeuroD4 (neuronal differentiation 4), which plays an important role in the development of neurons, significantly reduced the expression of SLC7A11 (cystine/glutamate transporter) and GPX4. This inhibition contributed to hindering the transport of glutamate into cells, reducing GSH synthesis, depletion of GPX4, and increased ferroptosis. Thus, the therapeutic effect of NeuroD4 was manifested by inhibiting the SLC7A11-GSH-GPX4 axis [191]. In another study, it was also shown that inhibition of GPX4 expression and activation of ferroptosis were activated by the combined use of cold atmospheric plasma and temozolomide [192].

GPX4 is also significantly expressed in lung cancer cells and could potentially become a target of anti-cancer drugs for the treatment of NSCLC cells [193]. Thus, the inducer of ferroptosis, erastin, reduces the radioresistance of NSCLC cells by inducing GPX4-mediated ferroptosis [194]. Besides. GPX4 can serve as a specific target of KLF11, a member of the Kruppel-type zinc finger transcription factor family, in the ferroptosis pathway [195]. Another study showed that the CREB transcription factor is an important component of the antioxidant system and plays an antioxidant role by stimulating GPX4 transcription, thereby inhibiting ferroptosis of lung adenocarcinoma cells [196].

Analysis of GPX4 expression in breast cancer cells revealed its significant expression in TNBC cells. At the same time, GPX4 can contribute to the malignant progression of cancer, since apoptosis, hypoxia, invasion, cell cycle, and DNA damage were significantly associated with GPX4 expression in TNBC [197]. In addition, GPX4 levels were significantly elevated in breast cancer tissue compared to normal tissue and were associated with cancer stages. In addition, GPX4 induces apoptosis by upregulating EGR1 (Early growth response-1), an early response gene involved in and involved in growth, differentiation, apoptosis, neurite growth, and wound healing. In the same work, it was found that a derivative of the natural product of parthenolide (DMOCPTL) is able to reduce the expression of GPX4, inducing its ubiquitination by direct binding to this glutathione peroxidase. Another study also described a similar regulation of GPX4 expression in cancer cells of TNBC. Thus, it was found that acetylcysteine, which demonstrated significant affinity for binding to GPX4, significantly reduced GPX4 protein levels by inducing ubiquitination. These events led to the activation of autophagically dependent ferroptosis in cells of TNBC [198]. It has also been found that in breast cancer cells, gankirin is able to prevent cellular ferroptosis through the gankirin/p53/SLC7A11/GPX4 signaling pathway [199]. Another study found that GPX4 is ubiquitinated by TNF receptor-associated factor 6 (TRAF6), which promotes its recognition by p62 and leads to its selective autophagic degradation [200]. Xenograft tumors from breast cancer patients with BRCA1 mutation (breast cancer susceptibility gene 1-deficient tumors) and PARPi resistance were also found to exhibit reduced GPX4 expression and high sensitivity to PARP and GPX4 coinhibition. These data suggest that BRCA1 regulates GPX4 transcription through its BRCT domain [201]. Zhang et al. found that resveratrol can inhibit GPX4 expression and suppress ferroptosis in TNBC cells by inhibiting the ERK1/2/SGK1/NEDD4L/GPX4 pathway in vitro and in vivo [202]. Other authors have also demonstrated that GPX4 activity in TNBC cells can be regulated by its ubiquitination. Thus, nobiletin-AKR1C1 promotes GPX4 degradation by enhancing its ubiquitination [203]. Another molecular mechanism for the suppression of GPX4 expression in MDA-MB-231 and BT-549 cancer cells, two TNBC cell lines, is the effect of simpevir on Nrf2/GPX4, which leads to ferroptosis [204]. To date, a large number of studies have been accumulated in which the therapeutic effect of any agent or drug comes down to the inhibition of GPX4 expression and activity in various models of breast cancer and the enhancement of ferroptosis [205,206,207,208,209,210,211,212,213,214,215,216,217,218,219]. Such regulation occurs through the inhibition of various signaling pathways, but the final target in them is GPX4.

On the other hand, there are a number of drugs that can enhance GPX4 expression. Thus, osteopontin was found to stimulate tumor growth and metastasis, as well as GPX4-mediated antilipid peroxidation in TNBC by activating the PI3k/Akt/mTOR pathway [220]. Another study also demonstrated that agents that promote TNBC progression also enhance GPX4 expression. Thus, RUNX1-IT1 (intronic transcript 1 of the RUNX1 gene) was shown to promote breast cancer carcinogenesis by blocking ferroptosis through increased GPX4 levels, and targeting the previously underestimated RUNX1-IT1/IGF2BP1/GPX4 regulatory axis [221]. Another mechanism of GPX4 expression regulation in breast cancer is its epigenetic modification. It was found that methyltransferase METTL16 regulates m6A methylation of GPX4, which enhances its expression, stimulates proliferation, and suppresses ferroptosis of breast cancer cells [222]. Furthermore, GPX4 transcription regulation in MDA-MB-231 and MCF7 cancer cells can be mediated by the interaction of zinc finger E-box homeobox 1 (ZEB1) with a region in the GPX4 gene that contains four E-box motifs (CANNTG). ZEB1 is a versatile transcription factor that can not only regulate EPT but also inhibit GPX4 transcription, which has a therapeutic effect on breast cancer metabolism [223].

GPX4 protein levels were higher in prostate cancer tissues with lymph node metastases than in tissues without metastasis. However, GPX4 levels gradually increased in metastatic cancer cells, which coincided with the expression levels of serum/glucocorticoid-regulated kinase 2 (SGK2) [224]. Based on these data, the authors suggested that GPX4 may be an important molecule downstream of SGK2, promoting ferroptosis. Complex regulation of GPX4 expression and HMGA2, a chromatin architectural protein, was documented to enhance oxidative stress and increase sensitivity to ferroptosis inducers. The authors found that PC3, DU145, and RWPE1 cells expressing high levels of endogenous HMGA2 protein demonstrated low or insignificant levels of GPX4 protein, which was associated with higher levels of lipid peroxides [225]. At the same time, in prostate cancer cells LNCaP, 22Rv1, C4-2B, MDAPCa-2b, low or moderate levels of HMGA2 and higher amounts of GPX4 and generally lower levels of lipid peroxides were recorded. It was also shown that HMGA2 does not alter the transcription of GPX4, but rather alters the transcription of SLC7A11 (cysteine/glutamate solute transporter family 7 member 11). There is also a wide range of other studies in which GPX4 acts as a target, and the suppression of the expression and activity of this protein contributes to the activation of ferroptosis in prostate cancer cells [226,227,228,229,230,231,232,233,234].

Thus, in virtually all cancer models of various etiologies, GPX4 acts as a target for anti-cancer action. In addition to the above-mentioned studies, there is a wide range of studies that have demonstrated the complex regulation of GPX4 expression through the activation of various signaling cascades, the final target of which is GPX4, which leads to ferroptosis of cancer cells [235,236,237,238,239,240,241,242,243,244,245,246,247,248,249,250,251,252,253,254,255,256].

However, there are virtually no studies proving the suppressive function of GPX4 in the regulation of carcinogenesis processes. After conducting a large-scale analysis of studies, we found that a suppressive role of GPX4 was established using pancreatic ductal adenocarcinoma as an example [257]. Thus, an analysis of overall survival showed that high GPX4 expression correlates with increased patient survival, whereas low GPX4 expression in combination with high expression of transmembrane protein 173 (TMEM173) increases patient mortality. Furthermore, it has been shown that high-iron diets or low GPX4 expression promote the development of KRAS-induced pancreatic tumors. GPX4 depletion or a high-iron diet increases the production and release of oxidized nitrogenous base, which promotes the subsequent accumulation and activation of macrophages with abnormal cytokine production, especially IL-6 and NOS2. IL-6 and NOS2 play an important role in all stages of PDAC and correlate with poor survival. Also, Cejas et al. found that GPX4 expression levels were reduced in poorly differentiated (grade 3) invasive ductal carcinoma of the breast, with the expression level gradually decreasing with increasing tumor grade from grade 1 to grade 3 [258]. The authors also found that decreased GPX4 expression correlated with decreased expression of the tumor suppressor p53. These results suggest that decreased GPX4 expression may be associated with poor prognosis in invasive ductal carcinoma of the breast.

**Figure 1:**
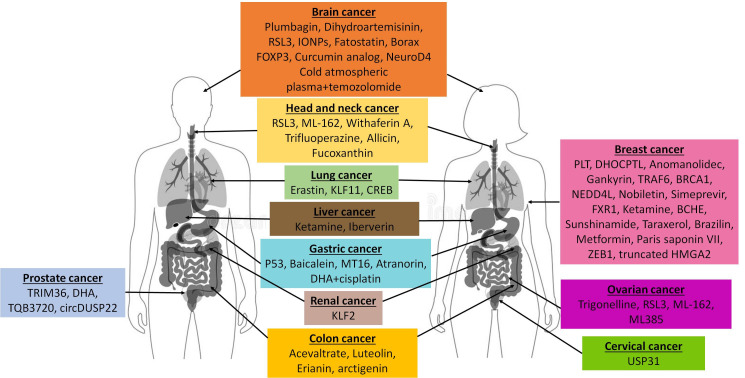
Main agents that are able to inhibit GPX4 expression and induce ferroptosis in various cancers. RSL3—RAS-selective lethal3; IONPs—iron oxide nanoparticles; FOXP3—Forkhead Box P3; NeuroD4—neuronal differentiation 4 transcription factor; KLF11—kruppel-type zinc finger transcription factor; PTL—parthenolide; DHOCPTL—derivative of natural parthenolide; TRAF6—TNF receptor associated factor; BRCA1—tumor suppressor breast cancer susceptibility gene 1; NEDD4L—neural precursor cell expressed developmentally downregulated gene 4-like; FXR1—fragile X mental retardation syndrome-related protein 1ragile X; BCHE—Boswellia carterii n-hexane extract; ZEB1—zink finger E-box binding homeobox 1; HMGA2—high mobility group A2;TRIM36—trigred motif 36; DHA—docosahexaenoic acid; TQB3720—androgen receptor antagonist; circDUSP22—circular RNA DUSP22; MT16—metallothionein; KLF2—Krüppel-like Factor 2; USP31—ubiquitin-specific peptidase 31.

Figure 1 shows the main agents that are able to inhibit GPX4 expression and induce ferroptosis in various cancers.

### 2.5. Glutathione Peroxidase 6 (GPX6)

GPX6 is a homolog of previously characterized proteins of the glutathione peroxidase family. GPx6 is a tetramer, a selenoprotein in humans and a non-Se enzyme in rodents. According to in situ hybridization, GPX6 is expressed in Bowman’s glands, where many components of olfactory mucus are synthesized and secreted. In addition, GPX6 has been shown to be expressed in mammalian tissues during the early stages of embryonic development [7]. The results obtained using confocal microscopy showed that SELENOV and GPX6 are cytoplasmic proteins that are not localized in mitochondria. Currently, there are few studies indicating the involvement of this human selenoprotein in the regulation of carcinogenesis. Some studies demonstrate low expression of GPX6 mRNA in various cancer cells [259,260,261].

At the same time, there are a number of studies indicating the regulation of GPX6 in gastric cancer. Thus, the results of the Kaplan–Meier plotter database analysis showed that increased GPX6 mRNA expression correlated with overall survival, which was statistically significant in patients with gastric cancer. The results of the Lauren classification showed that overall survival had good dynamics with increased GPX6 mRNA expression in patients with gastrointestinal cancer, diffuse gastric cancer and mixed gastric cancer [262]. In patients with negative human epidermal growth factor receptor 2 (HER2) expression, GPX1-6 mRNA expression was significantly correlated with overall survival in gastric cancer patients. Information on the role of GPX6 in the regulation of carcinogenesis is currently virtually nonexistent.

Studies have shown that GPX6 gene polymorphism is closely associated with the occurrence and classification of ovarian cancer [263].

High GPx6 mRNA expression was correlated with decreased overall survival in never-smoking patients with NSCLC [264].

## 3. Key Mechanisms Regulating the Expression Levels of Selenium-Containing GPXs in Various Cancers

Based on the above data, several key mechanisms influencing the expression of selenium (Se)-containing GPXs in various cancer cells can be identified. However, it is necessary to distinguish between the expression patterns of these glutathione peroxidases in intact cancer cells and the regulation of this expression by exposure of cancer cells to a known anti-cancer drug or potential anti-cancer compound.

### 3.1. Factors Affecting the Baseline Level of Glutathione Peroxidases in Various Cancer Cells

In intact cancer cells, the key events influencing glutathione peroxidase expression patterns are tissue-specific characteristics of cancer cells, which dictate not only the gene mutation profile specific to a particular cancer but also the activity of various processes that can generate varying levels of ROS and glutathione concentrations in cancer cells. Furthermore, the expression level of glutathione peroxidases may be directly dependent on the cellular concentration of Se, which is necessary for the biosynthesis of these enzymes.

The central dogma of cancer is that it arises in cells originating from various tissues due to the gradual accumulation of mutations in oncogenes and tumor suppressor genes. Comprehensive DNA profiling of tumors has revealed that, although there is some overlap, cancer genetics varies greatly depending on the tumor type and among individual patients, indicating that cancer is a personal disease of each patient’s tumor genome. Therefore, the genes encoding GPXs may also harbor a specific set of mutations: deletions, SNPs, and other abnormalities that affect the expression levels of these genes. It is known that certain genetic mutations (deletions, duplications, etc.) can lead to frameshifts and disrupt the synthesis of these proteins. It has been repeatedly shown that GPXs genes have SNPs in various tumor cells [265,266,267,268,269,270,271].

It is also well known that specific signaling pathways and the tumor microenvironment are activated in certain cancer cells, which, in particular, can affect the level of ROS, the amount of glutathione in these cells, and the activity of intracellular redox systems. Thus, it is well known that many types of tumors contain high levels of intracellular glutathione, which not only neutralizes oxidative damage caused by ROS, but also confers drug resistance to cancer cells. For example, patients with squamous cell carcinoma of the head and neck have a high ratio of disulfide glutathione to reduced glutathione (GSSG/GSH), as well as a high level of oxidative stress [51]; as another example, the level of GPX2 is sharply reduced in the highly metastatic breast cancer cell line PyMT2 compared to the low-metastatic tumor cell line PyMT1, which produced lower levels of ROS [89]. Given that the primary function of GPXs in cells is the reduction of hydrogen peroxide and other soluble hydroperoxides using glutathione, the balance of these compounds significantly influences GPXs levels in cancer cells.

Furthermore, Se concentrations in the tumor microenvironment can significantly influence basal GPX expression in cancer cells. Different GPXs are known to respond differently to Se deficiency. For example, GPX1 is known to occupy the lowest position in the selenoprotein hierarchy [271,272], meaning its expression is easily reduced by moderate Se deficiency, and its mRNA is degraded under these conditions. While maximum GPX3 expression can be observed at lower Se status [272,273], there are examples indicating that genetic variability in GPX genes, along with gender, can influence Se levels. For example, individuals with the GPX1 679T/T genotype, which is associated with an increased risk of developing certain cancers, have lower plasma Se levels than individuals with the GPX1 679C/C allele [273,274,275].

The above factors influencing basal GPX expression also determine the role of these enzymes in cancers of various etiologies: tumor suppressor or pro-oncogenic.

### 3.2. Fluctuations in GPX Expression Levels Depending on the Nature of the Agent Acting on Cancer Cells

When GPXs act as targets in the activation of signaling cascades aimed at inhibiting carcinogenesis, their expression can change dramatically, which is determined by the dichotomous nature of their role in these processes.

Such regulation can also occur at both the transcriptional and translational levels. To date, numerous examples of transcriptional regulation of GPX expression in various cancer cells have been presented. For example, GPX1 expression is known to be regulated at the transcriptional level by several transcription factors: PU.1, p53, NF-κB, AP-1, AP-2, ZNF143, and OREBP [38,39,40,41,42]. Fluctuations in GPX2 expression in various types of breast cancer cells depended on p53 status [92,93]. A large number of studies have been devoted to the influence of various miRNAs on GPX expression. For example, miR-92 in A549 lung cancer cells interacted with the 3′-untranslated region of GPX3 and inhibited its expression [156]. In glioblastoma cells, the transcription factor FOXP3 was shown to bind to five sites in the GPX4 promoter and directly regulate its expression, enhancing it [189].

Epigenetic regulation plays a significant role in regulating GPX expression. For example, the GPX3 promoter is particularly susceptible to hypermethylation; i.e., the addition of methyl groups to cytosine bases in DNA. This is often accompanied by GPX3 dysfunction, and therefore its expression is significantly reduced in many tumors [138,139,140,150,160].

Furthermore, the regulation of GPX expression can be controlled by post-translational degradation. For example, a derivative of the natural product parthenolide (DMOCPTL) is able to reduce GPX4 expression by inducing its ubiquitination through direct binding [197]. Another study also described a similar regulation of GPX4 expression in TNBC cells: acetylcysteine significantly reduced GPX4 protein levels by inducing ubiquitination in TNBC cells [198].

Alternatively, the expression of GPXs may depend on the expression of enzymes involved in the biosynthesis of these selenoproteins. For example, in hepatocellular carcinoma cells, GPX1 expression levels were directly dependent on the level of Se-binding protein 1 (SBP1), which enhanced GPX1 activity [66]. SBP1 and GPX1 were found to physically interact and, under conditions of oxidative stress, are capable of forming nuclear bodies and colocalizing. GPX1 is also able to suppress SBP1 transcription.

Thus, by analyzing these data, we can come to a conclusion about the complex mechanisms of regulation of GPXs expression in various cancer cells and tissues, which is carried out at different levels of regulation and determines the role of these important enzymes in carcinogenesis.

## 4. The Nature of the Expression of Se-Containing Thioredoxin Reductases in Cancers of Various Etiologies and Signaling Pathways with Their Participation

Selenocysteine-containing thioredoxin reductases (TXNRD/TrxR) are flavoproteins that play a key role in maintaining cellular redox homeostasis by catalytically reducing oxidized thioredoxin (Trx) using NADPH. In mammals, the family comprises three major selenocysteine (Sec)-containing isoforms: cytosolic TrxR1 (TXNRD1), mitochondrial TrxR2 (TXNRD2), and predominantly testicular TrxR3 (TGR/TXNRD3). Each isoform exhibits unique subcellular localization, substrate specificity, and is involved in distinct signaling pathways regulating apoptosis, proliferation, and the oxidative stress response [6,276]. In multiple cancer types, context-dependent overexpression of these enzymes promotes tumor progression, metastasis, and the development of therapy resistance, rendering them promising therapeutic targets.

### 4.1. Thioredoxin Reductase 1 (TrxR1/TXNRD1)

The thioredoxin system, a key component of which is thioredoxin reductase 1 (TrxR1, encoded by the *TXNRD1* gene), is a central regulator of cellular redox homeostasis. As a selenocysteine-containing flavoprotein, TrxR1 reduces oxidized thioredoxin (Trx) using NADPH, thereby maintaining the reductive environment necessary for the function of numerous signaling pathways, DNA synthesis, and antioxidant defense. Human TrxR1 is a homodimeric enzyme, with each subunit containing domains for FAD and NADPH binding, as well as a critically important C-terminal motif -Gly-Cys-Sec-Gly- that incorporates selenocysteine (Sec)—the 21st proteinogenic amino acid [277]. This selenocysteine residue is responsible for the enzyme’s high catalytic efficiency and its distinct susceptibility to a broad spectrum of inhibitors. Three isoforms of thioredoxin reductase have been identified in mammals: the cytoplasmic/nuclear TrxR1 (TXNRD1), the mitochondrial TrxR2 (TXNRD2), and the predominantly testicular TrxR3 (also known as TGR or TXNRD3) [278]. In the context of carcinogenesis (Table 4), the biological role of TrxR1 assumes a profoundly paradoxical character: from a protective suppressor in the early stages to a powerful driver of progression and resistance in advanced tumors. The primary function of TrxR1 is to maintain a reduced pool of Trx, which, in turn, regulates the activity of transcription factors (p53, NF-κB, HIF-1α), ensures the operation of the DNA repair system, and controls apoptotic signaling cascades.

Hyperexpression and hyperactivity of TrxR1 are common features of many aggressive malignant neoplasms. The oncogenic action of the TrxR1/Trx system is realized through a series of conserved mechanisms. A key mechanism is the suppression of apoptosis: reduced Trx directly binds to and inhibits apoptosis signal-regulating kinase 1 (ASK1), blocking the pro-apoptotic JNK/p38 cascades. In parallel, the system ensures the activation of transcriptional programs for survival and progression by maintaining the DNA-binding capacity of factors such as NF-κB and HIF-1α. This enhances the expression of genes involved in cell survival, proliferation, angiogenesis, and invasion. Furthermore, TrxR1 supports proliferation through the reduction of ribonucleotide reductase, a key enzyme for DNA synthesis, and promotes invasion and metastasis by regulating the activity of metalloproteinases and the epithelial–mesenchymal transition (EMT). An extremely important clinical consequence is the central role of TrxR1 in the development of therapy resistance. The enzyme functions as a powerful buffer, neutralizing oxidative stress induced by chemotherapeutic agents and ionizing radiation, while also supporting the function of DNA repair systems [12,278,279].

The specific manifestations of these universal mechanisms and their clinical significance vary depending on the tumor type. In breast cancer, TrxR1 functions as a definitive prognostic biomarker for poor outcomes and an oncogenic driver. A meta-analysis of data from over 13,000 patients revealed that high expression of the *Txnrd1* gene is associated with aggressive subtypes (HER2+, basal-like, luminal B), high histological grade, and negative estrogen and progesterone receptor status. Patients with high TrxR1 levels experience significantly earlier recurrences and metastasis [279]. It is interesting to note that TrxR1 exerts a dual influence on the therapeutic response. While it increases sensitivity to anthracyclines by facilitating redox cycling of the drug, it simultaneously enhances radioresistance. This finding underscores the necessity for personalized treatment approaches [279].

In colorectal cancer and other gastrointestinal carcinomas, elevated TrxR1 expression correlates with advanced stage, lymphogenous metastasis, and poor survival [12]. In addition to general mechanisms, metabolic adaptation plays a crucial role here: the TrxR1/Trx system enhances the pentose phosphate pathway through activation of G6PD, ensuring the production of NADPH and precursors for nucleotide synthesis under glucose-deficient conditions [280]. Furthermore, adaptation to hypoxia via stabilization of HIF-1α is also a key component [281].

For the group of head and neck squamous cell carcinomas (larynx-LSCC, esophagus-ESCC, oral cavity-OSCC), a consistent TrxR1 hyperexpression is characteristic, directly linked to aggressiveness and resistance. The mechanisms are integrated into the etiology, where chronic exposure to tobacco and alcohol induces oxidative stress, leading to activation of the Nrf2 factor and subsequent TrxR1 overexpression [282]. Universal pathways (NF-κB, HIF-1α, ASK1 inhibition) are also actively involved. TrxR1 maintains the activity of the EGFR/PI3K/Akt pathways, inhibits apoptosis, and ensures radioresistance [283]. Elevated enzyme levels are an independent prognostic marker for poor outcome [284,285,286]. This dependency renders TrxR1 a vulnerable therapeutic target for sensitization strategies. Co-administration of TrxR1 inhibitors (e.g., auranofin) with cisplatin or radiation exhibits a synergistic therapeutic effect in preclinical models [287].

The role of TrxR1 in hepatocellular carcinoma (HCC) presents a notable paradox, highlighting the profound context-dependency of its function. In clinical cohorts and a majority of preclinical models, TrxR1 exhibits oncogenic properties, with its overexpression correlating with vascular invasion, tumor recurrence, and acquired resistance to sorafenib [277,288]. Conversely, during the early stages of tumor initiation, TrxR1 exerts a crucial protective, tumor-suppressive function through its role in maintaining p53 activity. In a murine model of chemically induced hepatocarcinogenesis, hepatocyte-specific deletion of *Txnrd1* leads to a significant increase in tumor burden, characterized by elevated tumor incidence and multiplicity [289]. Thus, TrxR1 protects hepatocytes from cancer initiation, but promotes the progression of an already formed tumor.

In highly aggressive tumors such as glioblastoma (GBM) and neuroblastoma (NB), the TrxR1 system plays a pivotal role in maintaining redox homeostasis, suppressing apoptosis, and driving the development of therapy resistance [290]. However, the mechanistic context of this reliance differs between malignancies. In glioblastoma (GBM), TrxR1 overexpression is largely an adaptive mechanism driven by chronic oxidative stress in the hypoxic tumor microenvironment and in response to treatment. This renders TrxR1 a strategic target for circumventing the common resistance to both temozolomide and radiotherapy [291,292]. In neuroblastoma (NB), particularly in aggressive MYCN-amplified subtypes, TrxR1 overexpression is largely mediated by MYCN-driven oncogenic stress [293]. This establishes a unique therapeutic vulnerability, as the reliance on TrxR1 represents a synthetic lethal interaction. Consequently, TrxR1 inhibition demonstrates particularly high efficacy in this specific tumor subgroup [294,295].

**Table 4:** Data on the role and mechanisms of regulation of TXNRD1 in cancer of various etiologies.

**Types of Cancer with TXNRD1 as Tumor Marker**	**Mechanism of Cancer Progression**	**Ref.**
breast cancer	by the ASK1, NF-κB, HIF-1α, EMT	[278,279]
head and neck squamous cell carcinoma (HNSCC)	by Nrf-2, GFR/PI3K/Akt, ASK1	[282,283]
laryngeal squamous cell carcinoma (LSCC)	by Nrf-2, GFR/PI3K/Akt, ASK1	[285]
esophageal squamous cell carcinoma (ESCC)	by ASK1 inhibition, NF-κB and HIF-1α activation	[284,287]
gastric cancer	by through stabilization of HIF-1α, induction of angiogenesis (VEGF), enhancement of the pentose phosphate pathway through activation of G6PD, STAT3, EMT.	[280,281,296]
oral squamous cell carcinoma (OSCC)	by ASK1 inhibition, NF-κB activation	[286]
hepatocellular carcinoma (HCC)	ASK1 inhibition, NF-κB support, HIF-1α stabilization, oxidative stress neutralization	[277,288]
glioblastoma (GBM), neuroplastoma (NB)	suppression of apoptosis (ASK1), activation of transcriptional programs (NF-κB, HIF-1α), MYCN (NB)	[291,292,293,294]
colorectal cancer	by stabilization of HIF-1α, activation of the pentose phosphate pathway via G6PD, activation of invasion and metastasis (STAT3, EMT)	[12,280,281]
**Types of Cancer with TXNRD1 as Tumor Suppressor**	**Mechanism of Cancer Suppression**	**Ref.**
breast cancer	unknown	
head and neck squamous cell carcinoma (HNSCC)	unknown	
esophageal squamous cell carcinoma (ESCC)	unknown	
gastric cancer	unknown	
hepatocellular carcinoma (HCC)	by p53, APE1/Ref-1	[289]
low-grade gliomas (LGG)	unknown	
colorectal cancer	unknown	
glioblastoma (GBM), neuroplastoma (NB)	unknown	

Thus, thioredoxin reductase 1 (TrxR1) emerges as a central hub of redox regulation with a profoundly dualistic role in oncology. In established tumors across various tissue origins, it consistently functions as a driver of progression, metastasis, and therapy resistance through a set of conserved molecular mechanisms. Importantly, the context of its activity—whether etiological, oncogene-driven, or adaptive—shapes the specific disease manifestations and the potential efficacy of targeted inhibition. The universality of its oncogenic functions, coupled with the unique vulnerability of its selenocysteine-containing active site, establishes TrxR1 as a highly promising target for novel anti-cancer strategies aimed at disrupting the redox homeostasis of malignant cells.

### 4.2. Thioredoxin Reductase 2 (TXNRD2, TrxR2)

Thioredoxin reductase 2 (TXNRD2/TrxR2) is a selenocysteine-containing enzyme resident in the mitochondrial matrix and serves as the central component of the mitochondrial thioredoxin system. It functions to catalyze the NADPH-dependent reduction of mitochondrial thioredoxin 2 (Trx2). The critical importance of TrxR2 in preserving the redox homeostasis of this key bioenergetic compartment is highlighted by the embryonic lethality resulting from its complete genetic ablation in mice [297]. In cancer biology (Table 5), TXNRD2 activity exhibits a functional paradox. While it acts as a critical adaptive mechanism that promotes tumor cell survival, it simultaneously establishes a targetable biochemical dependency, creating an exploitable vulnerability unique to malignant cells [298].

In most solid tumors, the pro-oncogenic role of TXNRD2 is executed through a set of conserved mechanisms. Foremost among these is the direct inhibition of the intrinsic (mitochondrial) apoptotic pathway. TXNRD2, via its reduction of Trx2, facilitates the suppression of the pro-apoptotic kinase ASK1 and contributes to preserving outer mitochondrial membrane integrity, thus blocking the critical initiating event of cytochrome c release [299]. Simultaneously, TXNRD2 functions as the principal mitochondrial antioxidant defense system, scavenging reactive oxygen species (ROS) produced by oxidative phosphorylation. This protection not only prevents oxidative cell death but also maintains the bioenergetic capacity of mitochondria—a critical requirement for sustaining the high energy demands of uncontrolled proliferation [300]. These specific functions form the molecular basis for the broad role of TXNRD2 in driving therapy resistance. A key mechanism of action for numerous chemotherapeutic drugs and radiotherapy is the induction of lethal oxidative stress and the activation of apoptotic pathways—processes directly counteracted by TXNRD2 activity [301]. Furthermore, via its crosstalk with the glutathione antioxidant system, TXNRD2 contributes to ferroptosis protection by helping to maintain the activity of glutathione peroxidase 4 (GPX4), thereby introducing an additional mechanism of cellular resilience [302].

Nevertheless, the pathophysiological relevance of TXNRD2 transcends these universal mechanisms and is intricately linked to the distinct biology of specific cancers. A prime example is breast cancer, where TXNRD2 exhibits a potent tumor-promoting function, especially in aggressive molecular subtypes, by sustaining cancer stem cell traits and driving invasive behavior [303]. Intriguingly, this presents a therapeutic paradox. Although the TXNRD2 system generally promotes treatment resistance, its heightened activity can, in certain contexts, sensitize tumors to anthracycline-class chemotherapeutics. This occurs through its involvement in the futile redox cycling of these compounds, which exacerbates oxidative damage. Furthermore, pharmacological inhibition of TXNRD2 itself can increase cellular susceptibility to agents like doxorubicin [304]. In gastrointestinal carcinomas such as colorectal cancer (CRC), TXNRD2 frequently acts as a driver of multidrug resistance, particularly in tumors harboring wild-type KRAS [305]. In head and neck squamous cell carcinoma (HNSCC) arising from chronic exposure to tobacco and alcohol, TXNRD2 serves as a central adaptive node, enabling tumor survival under conditions of constant oxidative stress. It thereby functions as a direct mediator of resistance to first-line treatments such as cisplatin and radiotherapy [306].

**Table 5:** Data on the role and mechanisms of regulation of TXNRD2 in cancer of various etiologies.

**Types of Cancer with TXNRD2 as Tumor Marker**	**Mechanism of Cancer Progression**	**Ref.**
breast cancer	by suppression of mitochondrial apoptosis (inhibition of ASK1, prevention of cytochrome c release), support, protection from ferroptosis (via GPX4).	[303]
head and neck squamous cell carcinoma (HNSCC)	by ASK1, p38/JNK, Prx3, Prx5, GPX4	[306]
hepatocellular carcinoma (HCC)	by ASK1, p38/JNK, Prx3, Prx5, GPX4	[278,288]
glioblastoma (GBM)	by ASK1, Caspase-9, Caspase-3/7, Prx3, Prx5, APE1/Ref-1	[307]
colorectal cancer	By KRAS, suppression of mitochondrial apoptosis (ASK1 inhibition), protection from oxidative stress.	[305]
**Types of Cancer with TXNRD2 as Tumor Suppressor**	**Mechanism of Cancer Suppression**	**Ref.**
breast cancer	unknown	
gastric cancer	unknown	
bladder cancer	unknown	
glioblastoma (GBM)	unknown	
colorectal cancer	unknown	

The context-dependent duality of TXNRD2 is most strikingly evident in hepatocellular carcinoma (HCC). While in established tumors it unequivocally promotes an aggressive phenotype and resistance to sorafenib, during the initiation stage of carcinogenesis, as demonstrated in animal models, the basal activity of TXNRD2 and other selenoproteins may exert a protective, anti-initiation function by preventing malignant transformation [289]. The contrast in the enzyme’s role becomes even more striking when comparing glioblastoma and neuroblastoma. In glioblastoma, which maintains active mitochondrial respiration, TXNRD2 constitutes an unequivocally pro-oncogenic axis, critical for the survival of stem-like cells and therapy resistance [307]. In neuroblastoma, the role of TXNRD2 is critically modulated by MYCN oncogene status. High-risk, MYCN-amplified neuroblastomas exhibit a glycolytic phenotype and may show reduced dependence on classical mitochondrial antioxidant systems like TXNRD2. This altered metabolic state, however, establishes a unique therapeutic opportunity based on synthetic lethality: combined pharmacological inhibition of TXNRD2 and blockade of compensatory redox pathways could induce a lethal oxidative crisis selectively in these tumors [308].

Thus, the universal pro-oncogenic function of TXNRD2 in maintaining mitochondrial redox homeostasis positions it as a high-priority therapeutic target. Treatment strategies are based on the principle of synthetic lethality, exploiting the enzyme’s overexpression as a tumor-specific “Achilles’ heel.” Pharmacological inhibition using compounds such as auranofin or surfen triggers uncompensated mitochondrial stress in cancer cells, ultimately leading to cell death [309]. The most promising therapeutic avenue, however, involves rational combination strategies. These approaches co-administer TXNRD2 inhibitors with compounds that induce ferroptosis, deplete glutathione reserves, or with standard chemotherapeutics, thereby creating synergistic cytotoxicity and overcoming established mechanisms of drug resistance [310]. Ultimately, the efficacy of these personalized strategies will be dictated by careful consideration of the specific tumor context: the cancer type, its molecular and metabolic subtypes, and the tumor microenvironment. This context-aware precision is key to converting a fundamental cellular vulnerability into a targeted and effective anti-cancer therapy.

### 4.3. Thioredoxin Reductase 3 (TXNRD3, Thioredoxin-Glutathione Reductase or TGR)

Thioredoxin reductase 3 (TXNRD3) constitutes a distinct and relatively poorly characterized enzyme within the family of mammalian selenoprotein thioredoxin reductases. In contrast to the ubiquitously expressed cytosolic (TXNRD1) and mitochondrial (TXNRD2) isoforms, TXNRD3 exhibits a tightly restricted tissue distribution under normal physiological conditions. Its expression is most prominent in the testis, where it fulfills an essential function in sperm development and maturation [311]. Notably, similar to numerous testis-specific cancer/testis (CT) antigens, TXNRD3 is susceptible to ectopic reactivation in various malignancies, suggesting a potential contributory role in tumorigenesis [312].

The fundamental distinction of TXNRD3 lies in its hybrid molecular architecture, which combines functional domains of a thioredoxin reductase and a glutaredoxin. This unique structure confers dual enzymatic activity: the capacity to reduce both oxidized thioredoxin (Trx) and oxidized glutathione (GSSG). Consequently, TXNRD3 is positioned as a critical integrative hub linking the two principal cellular antioxidant systems [313]. Furthermore, in contrast to other family members, TXNRD3 possesses a distinctive dual subcellular distribution, being present in both mitochondria and the nucleus [314]. This strategic localization allows it to modulate redox signaling and homeostasis in two key compartments that govern fundamental cellular processes: bioenergetics, programmed cell death, and gene expression.

In cancer biology, this biochemical versatility enables TXNRD3 to act as a powerful facilitator of tumor adaptation. When cancer cells face severe oxidative/proteotoxic stress or when primary antioxidant pathways (e.g., TXNRD1 or GSH systems) are pharmacologically compromised, TXNRD3 can be upregulated to function as an essential compensatory mechanism. This supports cell viability and contributes to the development of treatment resistance. Notably, increased TXNRD3 expression has been documented in cancer cell lines with acquired resistance to targeted agents such as sorafenib and osimertinib [315].

The most compelling evidence elucidating a specific pro-oncogenic mechanism of TXNRD3 comes from studies on triple-negative breast cancer (TNBC). A genome-wide CRISPR/Cas9 screen identified TXNRD3 as a key factor mediating resistance of TNBC cells to the EGFR inhibitor erlotinib [316]. Mechanistically, TXNRD3 was found to act as a direct negative regulator of EGFR signaling. Its depletion (via genetic knockout or pharmacological inhibition by auranofin) results in reactive oxygen species (ROS) accumulation. This oxidative burst triggers redox-sensitive phosphorylation of EGFR at the key tyrosine 1068 residue and promotes its membrane localization. Therefore, under steady-state conditions, TXNRD3 constitutively suppresses EGFR pathway activity, which underlies the observed innate resistance to EGFR inhibitors like erlotinib. Inhibition of TXNRD3 reverses this state, leading to EGFR “activation” and re-sensitizing tumors to targeted therapy. This mechanism explains the powerful synergy observed for the auranofin-erlotinib combination in both cellular and animal models [316].

This specific mechanism perfectly illustrates the general therapeutic strategy targeting the thioredoxin system: inducing a pro-oxidant shift through reductase inhibition can disrupt the redox-dependent regulation of key oncogenic pathways and overcome resistance [317]. The multifunctionality of TXNRD3 renders it a challenging yet highly promising therapeutic target, as its inhibition has the potential to disrupt several compensatory antioxidant pathways simultaneously. The strategy of employing TrxR inhibitors (such as the FDA-approved drug auranofin) in sensitizing combination regimens is thus rationally justified [318].

Therefore, TXNRD3 is not a universal oncogenic driver but rather a specialized enzyme facilitating niche adaptation in cancer. Its critical importance is most evident in specific tumor subtypes (e.g., TNBC) or under substantial therapeutic stress, where it sustains survival by functioning as a “redox buffer.” Recent mechanistic insights, such as its role as a modulator of EGFR signaling and drug sensitivity, shift its status from a biological curiosity to a tractable therapeutic target for combating acquired resistance. Future exploration of TXNRD3 across diverse cancer types is likely to reveal additional context-specific redox vulnerabilities in aggressive malignancies.

In conclusion, the thioredoxin reductase family serves as a central regulatory nexus for cellular redox balance, playing a complex and context-dependent role in tumor biology. Their functional duality—ranging from tumor-suppressive in early carcinogenesis to tumor-promoting in advanced disease—highlights the necessity of a nuanced, context-aware approach in both fundamental research and translational oncology. The convergence of their widespread pro-tumorigenic activities and the unique, targetable selenocysteine catalytic center renders thioredoxin reductases a compelling target class for innovative therapeutic strategies designed to break therapy resistance and enhance clinical efficacy.

## 5. The Role of Subcellular Localization in the Functional Implementation of Se-Containing GPX and TXNRD

Data analysis suggests that the functional duality of Se-containing glutathione peroxidases and thioredoxin reductases is not limited to their tissue-specific expression or the stage of the tumor process. A critical level of regulation, largely determining their pro- or anti-tumor activity, is subcellular compartmentalization. Different isoforms of the same enzyme, generated through alternative splicing, the use of alternative translation start sites, or post-translational modifications, can localize to the cytosol, mitochondria, nucleus, or be secreted into the extracellular space. In each of these compartments, the enzyme interacts with a unique redox environment, a specific set of substrates, and protein partners, which fundamentally changes its role in redox signaling and carcinogenesis [6,165].

The most striking example of function depending on localization is demonstrated by GPX4. This enzyme exists in three isoforms generated by alternative translation initiation: cytosolic/nuclear (c-GPX4/n-GPX4), mitochondrial (m-GPX4), and spermatozoan nuclear (sn-GPX4) [165,166]. Cytosolic GPX4 is a key suppressor of ferroptosis. Its hyperexpression in glioblastoma, breast cancer, and non-small cell lung cancer cells is associated with resistance to chemotherapy, radiotherapy, and immune checkpoint inhibitors [178,193,197]. The mitochondrial isoform, in contrast, localizes to the inner mitochondrial membrane, where it reduces cardiolipin hydroperoxides [319]. This prevents cytochrome c release and inhibits the initiation of the mitochondrial pathway of apoptosis [167,198]. Thus, m-GPX4 performs an anti-apoptotic function that is mechanistically independent of ferroptosis. In certain models of drug resistance (e.g., to doxorubicin), a selective increase specifically in the mitochondrial pool of GPX4 is observed [201,202]. Consequently, simply assessing the total expression level of GPX4 is insufficient for prognosis; the balance of its isoforms must be taken into account.

For GPX1, the possibility of nuclear translocation has also been demonstrated. Normally, GPX1 is primarily a cytosolic enzyme; however, under conditions of hypoxia and oxidative stress, particularly in glioblastoma cells, it is found in the nucleus and in exosomes [70,71]. Nuclear GPX1 protects genomic DNA from damage induced by radiotherapy and cisplatin, representing an additional mechanism of multidrug resistance. Exosomal GPX1 can be transferred from resistant cells to sensitive ones, spreading resistance throughout the tumor population [70]. Thus, the redistribution of GPX1 between compartments serves as a mechanism for tumor cell adaptation to stressful conditions.

GPX3 is a unique member of the family, functioning primarily as an extracellular enzyme secreted by epithelial cells of the kidneys, thyroid gland, and lungs [117,126]. Suppression of GPX3 expression due to promoter hypermethylation, frequently observed in gastric, esophageal, and colorectal cancers, leads to the accumulation of extracellular H_2_O_2_ in the tumor microenvironment, which stimulates invasion and epithelial–mesenchymal transition [138,140,150]. Paradoxically, in several aggressive tumors (ovarian cancer, triple-negative breast cancer), intracellular hyperexpression of GPX3 is registered, which protects tumor cells in ascitic fluid and increases resistance to cisplatin [145,146,161]. Thus, the loss of the secreted form of GPX3 contributes to progression in the early stages, whereas the acquisition of intracellular expression supports the survival of metastatic cells.

For TXNRD1, the existence of nuclear splice variants is critically important. In addition to the canonical cytosolic isoform, the *TXNRD1* gene encodes variants (v2, v5, and others) that lack the N-terminal mitochondrial signal but contain a nuclear localization signal [6,278]. Cytosolic TXNRD1 reduces thioredoxin-1 (Trx1), inhibits ASK1 kinase, supports ribonucleotide reductase activity, and mediates the classic pro-oncogenic effects described in Section 4.1 and Section 4.2 [12,279]. Nuclear isoforms of TXNRD1 reduce the nuclear pool of Trx1, which serves as a critical cofactor for transcription factors such as NF-κB, HIF-1α, p53, and the Ref-1/AP-1 complex [320]. Activation of these transcriptional programs enhances angiogenesis, proliferation, and tumor cell survival. The expression of nuclear TXNRD1 variants is elevated in aggressive subtypes of breast cancer, glioblastomas, and colorectal cancer, and correlates with an unfavorable prognosis [279,292].

Unlike TXNRD1, TXNRD2 is an exclusively mitochondrial enzyme imported into the matrix, where it reduces mitochondrial thioredoxin-2 (Trx2) and glutaredoxin-2 (Grx2) [297,299]. The pro-oncogenic function of TXNRD2 is mediated through the suppression of the mitochondrial apoptotic pathway (inhibition of ASK1, prevention of cytochrome c release) and the neutralization of ROS generated during oxidative phosphorylation [300,321]. This function is critically important for tumors with active respiration (glioblastoma, melanoma), whereas in glycolytic tumors (e.g., MYCN-amplified neuroblastoma), the dependency on TXNRD2 is reduced, creating a vulnerability based on synthetic lethality [322].

The most complex compartmentalization is characteristic of TXNRD3 (TGR). This enzyme possesses unique dual localization, being present in both mitochondria and the nucleus [312,314]. Mitochondrial TXNRD3 performs a compensatory function in the context of TXNRD2 deficiency, maintaining redox homeostasis and contributing to resistance against targeted agents (sorafenib, osimertinib) [315]. Nuclear TXNRD3 has been identified as a negative regulator of the epidermal growth factor receptor (EGFR). In the nucleus, TXNRD3, by reducing Trx1, suppresses EGFR phosphorylation at Tyr1068 and its membrane translocation, underlying the intrinsic resistance of triple-negative breast cancer cells to EGFR inhibitors (erlotinib) [316]. Inhibition of TXNRD3 with auranofin simultaneously blocks both pools, which explains the potent synergy of the auranofin and erlotinib combination.

Thus, the subcellular localization of Se-containing GPXs and TXNRDs constitutes an independent level of regulation determining their oncogenic or tumor-suppressive potential. Neglecting compartment-specificity may lead to contradictory results when interpreting clinical data and the prognostic significance of these enzymes. From a practical standpoint, considering localization opens new avenues for “second-generation” targeted therapy: the development of inhibitors that selectively block the nuclear import of TXNRD1 or the degradation of cytosolic GPX4 could achieve an antitumor effect without the complete suppression of the physiological functions of these enzymes in healthy tissues. The generation of antibodies capable of differentiating between nuclear and cytosolic isoforms of TXNRD1 represents a promising direction for the advancement of immunohistochemical diagnostics.

## 6. The Evolutionary Shift from Tumor Suppressor to Oncogene During Cancer Progression

The dualistic roles of GPXs and TXNRDs are not merely a matter of different cancer types but represent a dynamic, context-dependent shift that occurs during the natural history of a tumor. This functional evolution is critical for resolving the “expression paradox” and can be conceptualized across three key phases of carcinogenesis. During the tumor initiation phase, the primary role of Se-containing antioxidant enzymes such as GPX1 and TXNRD1 is unequivocally tumor-suppressive. By efficiently neutralizing endogenous and exogenous reactive oxygen species, they prevent oxidative damage to DNA, lipids, and proteins, thereby reducing the mutagenic load that drives malignant transformation. The protective role of TXNRD1 against chemically induced hepatocarcinogenesis, where its deletion promotes tumor initiation, serves as a prime example [289]. Similarly, the loss of GPX3 expression via promoter hypermethylation is recognized as an early event in cancers of the esophagus, stomach, and colon, facilitating the accumulation of further genetic and epigenetic abnormalities [138,140,150]. At this stage, maintaining high expression of these enzymes is beneficial and anti-carcinogenic. However, once malignant transformation is established, the selective pressure on the tumor cell reverses the role of these enzymes. Cancer cells face intrinsically high oxidative stress due to oncogene activation (e.g., *MYC*, *KRAS*), metabolic reprogramming, and hypoxia. In this context of tumor progression, they “hijack” the antioxidant machinery. GPX1, GPX2, and TXNRD1, which were once protective, are now overexpressed as part of an adaptive survival program. Their function shifts from preventing mutation to promoting proliferation, inhibiting apoptosis, and driving invasion. For instance, GPX2 is upregulated in many tumors to support growth and inhibit apoptosis downstream of oncogenic pathways like Wnt/β-catenin [83,86]. TXNRD1, through its nuclear isoforms, activates pro-survival transcription factors like NF-κB and HIF-1α, directly fueling angiogenesis and metastasis [279,291]. The same biochemical activity—reducing peroxides and maintaining a reducing environment—is beneficial for a normal cell but is exploited by a cancer cell to tolerate its own oncogenic stress and resist cell death, representing the essence of “non-oncogene addiction.” Finally, in advanced, metastatic, and therapy-resistant cancers, the dependency on specific selenoproteins becomes even more pronounced. This stage reveals the ultimate therapeutic paradox: the same enzyme that was a tumor suppressor in a normal cell becomes an exploitable vulnerability in a cancer cell. Cancer stem cells and drug-tolerant persister cells, which are responsible for relapse, are often exquisitely dependent on GPX4 to evade ferroptosis [176]. Similarly, aggressive tumors with high metastatic potential rely on TXNRD1 to maintain a reduced environment that supports invasion and epithelial–mesenchymal transition [12]. This heightened dependency creates a therapeutic window. Inhibiting GPX4 (e.g., with RSL3) or TXNRD1 (e.g., with auranofin) can selectively kill these otherwise resistant cancer cells, while normal cells, with lower baseline oxidative stress and multiple redundant pathways, may be less affected. Thus, the journey of these enzymes from tumor suppressors in normal tissue to oncogenic drivers in established tumors and finally to therapeutic targets in advanced disease illustrates a fundamental principle of redox biology in cancer: context is everything. This evolutionary perspective is critical for interpreting the “expression paradox” and for designing rational, stage-specific therapeutic interventions. This evolutionary trajectory from tumor suppressor to oncogenic driver and finally to therapeutic target is not a uniform process but is highly isoform-specific. A comprehensive summary of these functional specializations, including the predominant roles and key mechanisms for each GPX and TXNRD isoform across different cancer contexts, is presented in Table 6.

## 7. Conclusions

This review demonstrates that the families of selenium-containing glutathione peroxidases (GPXs) and thioredoxin reductases (TXNRDs) are not static entities but rather dynamic, context-dependent molecular switches in oncogenesis. Their biological function in tumor cells is characterized by pronounced duality that evolves during the multistep process of carcinogenesis. By framing their function within this stage-specific model—from tumor-suppressive gatekeepers during initiation to pro-oncogenic drivers during progression and finally to targetable vulnerabilities in advanced disease—we can begin to resolve the apparent ‘expression paradox.’ This evolutionary perspective clarifies why the same enzyme can have opposing roles and underscores the necessity of moving beyond simple expression analysis toward a more nuanced, context-aware interpretation. This functional duality is determined by a complex multi-level regulatory system, including tissue-specific transcription factors (p53, NF-κB, NRF2), epigenetic modifications (particularly hypermethylation of the *GPX3* promoter), post-transcriptional control via microRNAs, post-translational modifications, and critically, subcellular compartmentalization. Oncogenic effects are primarily realized through the maintenance of redox homeostasis, suppression of apoptosis, stimulation of proliferation and invasion, and the formation of resistance to therapeutic interventions. Conversely, suppressor activity is often associated with the induction of apoptosis and cell cycle arrest, as well as the inhibition of key oncogenic signaling pathways.

A special role is played by GPX4, whose primary function is to prevent ferroptosis—a form of iron-dependent programmed cell death. Inhibition of GPX4 represents one of the most promising strategies for overcoming resistance in tumors of various origins. Similarly, the overexpression of cytosolic TXNRD1 and mitochondrial TXNRD2 is a common feature of aggressive cancers and is directly correlated with poor prognosis, making them attractive pharmacological targets. Nevertheless, progress in this field is hindered by several limitations. Substantial discrepancies in experimental data, particularly regarding GPX1–3, often stem from high tumor heterogeneity and differences in the model systems used. The role of certain isoforms, such as GPX6 and TXNRD3, remains poorly understood. The significant gap between abundant preclinical data and their clinical validation presents a serious challenge.

The fundamental duality of Se-containing enzymes discussed above is directly reflected in a long-standing and clinically significant controversy regarding the use of selenium-containing supplements in cancer patients. On one hand, selenium is an essential micronutrient, and its deficiency in several epidemiological studies has been associated with an increased risk of cancer development and an unfavorable prognosis, presumably due to impaired synthesis of protective selenoproteins such as GPX and TXNRD [271,323]. This provides a rationale for supplementation, particularly in selenium-deficient populations. On the other hand, interventional clinical trials have yielded conflicting, and often concerning, results. The pivotal SELECT (Selenium and Vitamin E Cancer Prevention Trial) study demonstrated that supplementation with selenium-containing supplements (in the form of selenomethionine) does not prevent prostate cancer and may even potentially increase the risk of developing type 2 diabetes mellitus with long-term use [R1]. More importantly for oncology, some studies suggest that while low doses of selenium may be beneficial, high doses or the use of specific chemical forms (e.g., sodium selenite) may exert pro-oxidant and antitumor effects; however, their therapeutic window is narrow, and toxicity remains a concern [14,16]. The outcome appears to depend on a complex interplay of factors, including the patient’s baseline selenium status, the chemical form of selenium administered, genetic polymorphisms in selenoprotein genes (such as the GPX1 rs1050450 variant discussed in Section 2.1), and, critically, the type and stage of cancer [48,265]. For instance, in patients with established aggressive tumors that overexpress oncogenic selenoproteins like GPX2 or TXNRD1, additional selenium intake as a substrate could theoretically fuel tumor progression and the development of drug resistance, rather than combating them [12,90]. This clinical controversy is not coincidental but rather a direct consequence of the described phenomenon of the context-dependent duality of selenoproteins. It underscores the central thesis of our review: a universal, “one-size-fits-all” approach to selenium supplementation is untenable. Future clinical strategies must move beyond indiscriminate supplementation towards personalized, biomarker-guided approaches. Assessing a patient’s selenoprotein expression profile, tumor type, and genetic background could help identify those who would benefit from selenium repletion versus those for whom it could be potentially harmful, thereby resolving this long-standing clinical dilemma.

A critical and as yet insufficiently addressed limitation is the potential toxicity associated with systemic pharmacological inhibition of GPXs and TXNRDs. Both enzyme families are not merely oncogenic drivers in malignant tissues but fulfill essential cytoprotective functions in normal physiology. GPX1 and GPX4 are ubiquitously expressed and protect vital organs—including the liver, kidney, brain, and heart—from oxidative damage. Mitochondrial TXNRD2 is indispensable for cardiomyocyte survival and hematopoiesis, as evidenced by the embryonic lethality of its genetic ablation. Likewise, GPX4 contributes to sperm maturation, and its inhibition may impair male fertility. Consequently, the therapeutic window for systemically administered small-molecule inhibitors of these enzymes is likely to be narrow, and nonspecific toxicity remains a major translational hurdle.

Addressing this challenge requires the development of rational, context-aware therapeutic strategies rather than broad systemic suppression. Several complementary approaches hold promise. First, nanoparticle-based drug delivery systems (e.g., selenium- or iron-oxide-based nanocarriers) can facilitate tumor-selective accumulation of GPX/TXNRD inhibitors, thereby sparing normal tissues. For example, our recent work demonstrates that not only the composition but also the morphology of selenium nanoparticles (spheres vs. nanorods) critically influences their anti-cancer efficacy by differentially regulating Ca^2+^ signaling and ER stress pathways, highlighting the importance of nanocarrier design [324]. Second, the principle of synthetic lethality may be exploited to identify tumor subpopulations with inherent hypersensitivity to selenoprotein inhibition—such as BRCA1-deficient cancers exhibiting dependency on GPX4, or MYCN-amplified neuroblastomas vulnerable to TXNRD blockade—allowing for lower, less toxic doses. Third, combination regimens that pair subtoxic concentrations of GPX/TXNRD inhibitors with ferroptosis inducers, glutathione-depleting agents, or immune checkpoint blockers may achieve synergistic antitumor efficacy while minimizing off-target effects. Fourth, intermittent dosing schedules could permit recovery of physiological selenoprotein pools in normal tissues between treatment cycles. Thus, the future clinical translation of GPX- and TXNRD-targeted therapies lies not in their use as single agents administered at maximally tolerated doses, but in their rational integration into biomarker-driven, combination-based, and pharmacodynamically controlled regimens.

In conclusion, selenium-containing antioxidant enzymes, as central hubs of cellular redox regulation, open new avenues for the development of innovative anti-cancer strategies. The challenge of toxicity is real, but it is not insurmountable. A deeper understanding of the context-specific roles of each isoform, combined with advances in targeted drug delivery and synthetic lethality screening, can transform these fundamental biochemical vulnerabilities into effective and safe cancer treatments.

## Figures and Tables

**Table 1:** Data on the role and mechanisms of regulation of GPX1 in cancer of various etiologies.

**Types of Cancer with GPX1 as Tumor Marker**	**Mechanism of Cancer Progression**	**Ref.**
breast cancer	by the transcription factor TFAP2C (AP-2γ)	[43,44,45,46]
renal cell carcinoma (ccRCC)	unknown	[50]
head and neck squamous cell carcinoma (HNSCC)	by chemokine CXC motif ligand 16 (CXCL16)	[51,52,53,54]
laryngeal squamous cell carcinoma (LSCC)	unknown	[56]
esophageal squamous cell carcinoma (ESCC)	by vitamins D/NF-κB/GPX1 axis, by MMP2 and uPA	[57,58,59,60,61]
gastric cancer	by HGF NF-κB, PI3K/Akt and uPA	[62]
oral squamous cell carcinoma (OSCC)	unknown	[64]
salivary adenoid cystic carcinoma (SACC)	by NF-κB and uPA	[65]
hepatocellular carcinoma (HCC)	by SBP1	[66]
glioblastoma (GBM), neuroplastoma (NB)	by POU (NONO)	[7,8,9,10,11,12,13,14,15,16,17,18,19,20,21,22,23,24,25,26,27,28,29,30,31,32,33,34,35,36,37,38,39,40,41,42,43,44,45,46,47,48,49,50,51,52,53,54,55,56,57,58,59,60,61,62,63,64,65,66,67,68,69,70,71]
colorectal cancer	by PCNA	[72,73,74,75]
**Types of Cancer with GPX1 as Tumor Suppressor**	**Mechanism of Cancer Suppression**	**Ref.**
breast cancer	by the transcription factor TFAP2C (AP-2γ)	[48,49]
head and neck squamous cell carcinoma (HNSCC)	unknown	[51]
esophageal squamous cell carcinoma (ESCC)	by vitamins D/NF-κB/GPX1 axis, by MMP2 and uPA	[58]
gastric cancer	by promoter methylation	[63]
hepatocellular carcinoma (HCC)	by Se-binding protein 1	[66]
low-grade gliomas (LGG)	unknown	[69]
colorectal cancer	by PCNA	[73]
pancreatic ductal adenocarcinoma (PDAC)	by Akt/GSK3β/Snail signaling axis	[76]

**Table 2:** Data on the role and mechanisms of regulation of GPX2 in cancer of various etiologies.

**Types of Cancer with GPX2 as Tumor Marker**	**Mechanism of Cancer Progression**	**Ref.**
breast cancer	by p53	[92,93]
lung cancer	by ROS, MMP1, miR-325-3p	[94]
	by PI3K/AKT/mTOR	[84]
head and neck squamous cell carcinoma (HNSCC)	by ROS, NRF2/GPX2/NOTCH3	[114]
bladder cancer	unknown	[113]
prostate cancer castration-resistant cancer	by Wnt/β catenin/EMT	[86]
	unknown	[81]
gastric cancer	by ROS, ER-stress	[105]
hepatocellular carcinoma (HCC)	by ROS, β-catenin/TCF4	[83]
	by ER-stress	[102]
glioblastoma (GBM)	by CXCR1, CXCR2, XCR1	[96]
colorectal cancer	unknown	[108,109]
**Types of Cancer with GPX2 as Tumor Suppressor**	**Mechanism of Cancer Suppression**	**Ref.**
breast cancer	by Notch, NFkb, ROS/HIF1α/VEGFA	[89]
gastric cancer	by ROS, KYNU-Kyn-AhR	[104]
bladder cancer	unknown	[111,112,113]
glioblastoma (GBM)	unknown	[100]
colorectal cancer	by COX-2	[108]

**Table 3:** Data on the role and mechanisms of regulation of GPX3 in cancer of various etiologies.

**Types of Cancer with GPX3 as Tumor Marker**	**Mechanism of Cancer Progression**	**Ref.**
gastric cancer	by DUBR/miR-502-3p/GPX3 by increasing the infiltration of tumor immune cells, enhancing the expression of immune checkpoints, and reducing sensitivity to chemotherapeutic drugs	[142,143]
ovarian tumor	by regulating expression of GDF15	[145]
	by manipulating the extracellular redox environment	[146]
	by increasing resistance to cisplatin	[147]
breast cancer	by GPX3-TGFB1-ZEB2	[161]
**Types of Cancer with GPX3 as Tumor Suppressor**	**Mechanism of Cancer Suppression**	**Ref.**
esophageal squamous cell carcinoma	by suppress proliferation, metastasis, and MMP-9 expression by deactivating the FAK/AKT	[138,139]
gastric cancer	by Wnt/JNK	[144]
colorectal cancer	increased cell sensitivity to oxaliplatin and cisplatin	[150]
	by regulating cholesterol levels	[151]
lung cancer	by MKP3-Erk-NF-κB-cyclin B1	[152]
	by suppression miR-665	[154]
	by suppression miR-196a	[155]
breast cancer	by suppression miR-324-5p	[158]
	unknown	[159]
	unknown	[160]
papillary thyroid cancer	by Wnt/β-catenin	[162]
	by interacting with JUN	[163]
	by suppression miR-146b-5p	[164]

**Table 6:** Key Features and functional specialization of selenium-containing GPX and TXNRD isoforms in cancer.

Isoform	Major Tissue Distribution (Normal)	Primary Subcellular Localization	Key Oncogenic Role(s)/Predominant Function in Cancer	Tumor Suppressor Role(s)/Context of Loss	Key Mechanism(s)
GPX1	Ubiquitous	Cytosol, nucleus, exosomes	Progression, therapy resistance (GBM, breast, CRC, ESCC, RCC, LSCC, OSCC, SACC, gastric, HCC). Protects from oxidative stress, promotes invasion, and confers resistance to therapy (e.g., cisplatin, radiation).	Tumor suppressor (PDAC, gastric, some ESCC, some breast). Inhibits EMT via Akt/GSK3β/Snail in PDAC. Loss linked to promoter methylation (gastric) or polymorphisms (breast).	The “Hierarchy” and Duality Enzyme: Lowest in selenoprotein hierarchy, highly sensitive to Se status. Nuclear/Exosomal translocation drives resistance. Its role is highly dichotomous, varying greatly by cancer type.
GPX2	GI tract, liver, mammary gland	Cytosol	Oncogene (lung, gastric, HCC, prostate, HNSCC, GBM, CRC). Promotes proliferation, EMT, metastasis. Drives resistance (e.g., lenvatinib, cisplatin).	Context-dependent suppressor (breast, bladder). Loss linked to aggressive subtypes and poor prognosis in breast and bladder cancer.	The “GI/Oncogenic” GPX: Strong pro-oncogenic focus in most solid tumors. Can induce metabolic reprogramming (KYNU-AhR, lipid metabolism) and is often a target of NRF2, p53, and Wnt/β-catenin.
GPX3	Kidney (major source), thyroid, lung, epididymis	Secreted (extracellular), plasma	Context-dependent oncogene (ovarian, TNBC, gastric). Supports survival in ascites (ovarian), promotes resistance to cisplatin (ovarian, TNBC), drives invasion via GDF15 (ovarian) and TGFB1-ZEB2 (TNBC).	Classical tumor suppressor (esophageal, gastric, lung, thyroid, CRC, breast). Silenced by promoter hypermethylation. Loss promotes proliferation, metastasis, and invasion.	The “secreted/epigenetic” GPX: Main extracellular peroxidase. Silencing via hypermethylation is a common event. Dual role is stark: lost in early stages of many cancers, but can be hijacked for survival in advanced/metastatic contexts (e.g., ascites).
GPX4	Ubiquitous	Cytosol (c-GPX4), mitochondria (m-GPX4), nucleus (n-GPX4)	Master suppressor of ferroptosis, therapy resistance. Highly expressed in many cancers (TNBC, lung, prostate, GBM, HCC). Protects from lipid peroxidation, driving resistance to chemo-, radio-, and immunotherapy.	Potential suppressor (PDAC, breast). Low expression in PDAC promotes tumorigenesis via STING. Downregulation in poorly differentiated breast cancer.	The “ferroptosis” gatekeeper: The only GPX that reduces complex lipid hydroperoxides. Isoform-specific localization dictates function: m-GPX4 inhibits apoptosis, c-GPX4 inhibits ferroptosis. Primary target for novel anti-cancer drugs.
GPX6	Olfactory epithelium, embryonic tissues	Cytoplasm	Poorly characterized. Low expression in various cancers. Increased mRNA correlates with poor survival in NSCLC and some gastric cancer subtypes.	Poorly characterized. Increased mRNA correlates with better survival in other gastric cancer subtypes.	The “enigmatic” GPX: Human selenoprotein, but not in rodents. Its role in cancer is still largely unexplored and appears complex based on preliminary data.
TXNRD1	Ubiquitous	Cytosol, nucleus (splice variants)	Key oncogenic driver, progression, metastasis. Supports proliferation, inhibits apoptosis (ASK1), activates pro-survival TFs (NF-κB, HIF-1α), drives EMT, and confers therapy resistance.	Protective in initiation (HCC). Loss in early stages promotes chemically induced hepatocarcinogenesis, likely via p53 dysregulation.	The “cytosolic redox hub”: Maintains reduced Trx1, linking antioxidant defense to DNA synthesis and transcription. Nuclear variants activate pro-tumorigenic gene programs.
TXNRD2	Ubiquitous	Mitochondria (matrix)	Pro-survival, therapy resistance. Inhibits intrinsic apoptosis (ASK1, cytochrome c release). Scavenges mitochondrial ROS from OXPHOS, maintains bioenergetics. Supports cancer stem cell traits and therapy resistance.	Likely protective in initiation (HCC). Inference from TXNRD1 studies; not directly demonstrated for TXNRD2 in the provided text.	The “mitochondrial guardian”: Dedicated antioxidant for mitochondrial Trx2. Critical for tumors with high OXPHOS dependency. This dependency creates a potential synthetic lethality opportunity.
TXNRD3	Testis (predominant)	Nucleus and mitochondria	Adaptive resistance, redundancy. Acts as a compensatory mechanism when primary antioxidant pathways are inhibited (e.g., resistance to sorafenib, osimertinib). Supports survival of persister cells.	Context-specific tumor suppressor? (TNBC). Constitutively suppresses EGFR signaling; its inhibition paradoxically activates EGFR and sensitizes to erlotinib.	The “hybrid/redundancy” enzyme: Dual thioredoxin and glutathione reductase activity. Dual localization allows it to act as a “redox buffer” in both nucleus and mitochondria, driving niche adaptation and resistance.

## Data Availability

No new data were created or analyzed in this study. Data sharing is not applicable to this article.
